# Enhancing sustainable innovation through collaborative knowledge absorption: Insights from the development of a hybrid marine engine

**DOI:** 10.1371/journal.pone.0346929

**Published:** 2026-04-15

**Authors:** Jingjing Ge, Sandra Hasanefendic, Bart Bossink

**Affiliations:** Department of Breakthrough Tech Innovation, Faculty of Science, Vrije Universiteit Amsterdam, Amsterdam, The Netherlands; Bonn-Rhein-Sieg University of Applied Sciences: Hochschule Bonn-Rhein-Sieg, GERMANY

## Abstract

Rapid technological advancements, characterized by uncertainties, together with the growing imperative for environmental sustainability, are continuously reshaping markets and business strategies. These developments necessitate integrating sustainable innovation with effective knowledge absorption to promote technological progress. While prior research has primarily examined drivers of sustainability at the macro level, comparatively limited attention has been given to the processual and micro-level dynamics through which sustainable innovation unfolds. As a result, the underlying mechanisms, individual actions, and collaborative knowledge processes that shape sustainable innovation remain insufficiently theorized and empirically explored. To address this gap, this study draws on effectuation theory and absorptive capacity to conceptualize sustainable innovation as an iterative, stakeholder-driven process in which knowledge is acquired, assimilated, transformed, and exploited. Empirically, the study adopts an in-depth case analysis of the development of a hybrid marine engine – i.e., a propulsion system for vessels that combines a conventional combustion engine with electric propulsion from batteries – to refine this framework. The results identify five distinct phases of the sustainable innovation process, each influenced by specific dimensions of individuals’ collaborative knowledge absorption. This study contributes to the sustainability and innovation literature by integrating insights from effectuation, knowledge absorption, and entrepreneurial behavior into a structured framework. In doing so, it provides both theoretical advancement and practical guidance for policymakers and business practitioners seeking to foster sustainable innovation in technology-driven contexts.

## 1 Introduction

Rapid technological advancements – characterized by heightened uncertainties [1], escalating climate change challenges, and a global shift toward balancing economic growth with environmental sustainability [2] – are profoundly reshaping markets and business strategies. In response, firms are increasingly required to integrate innovative processes, products, and services with sustainable innovation strategies [3]. Achieving such integration requires a collaborative approach that involves diverse specialists [4], fosters scientific decision-making, and encourages iterative experimentation [5,6]. Central to this process is knowledge absorption, which underpins technological progress and innovation outcomes [7,8].

At the individual level, knowledge absorption has emerged as a critical determinant of sustainable innovation [9–11]. It plays a pivotal role in identifying and leveraging technology-driven opportunities [12,13], while simultaneously fostering sustainability-driven advancements [14,15]. Although existing research has examined sustainability-related innovation through lenses such as eco-innovation, environmental innovation, and sustainable innovation orientation [16–18], the predominant focus has been on identifying internal and external determinants or drivers of sustainable outcomes [19,20–22]. Consequently, comparatively limited attention has been paid to the processual and micro-level dynamics through which sustainable innovation is enacted, leaving the underlying mechanisms, individual actions, and collaborative knowledge processes insufficiently theorized and empirically examined [23–25].

This limitation is particularly salient given that scaling up sustainable innovation, which entails the integration of social, environmental, and economic goals, is inherently complex and multifaceted [26,27]. Such complexity necessitates the active involvement of multiple stakeholders, particularly individuals who collaboratively generate, share, and apply knowledge to create value aligned with these integrated goals [28,27,21]. Despite the recognized importance of collaboration and knowledge-related processes, the role of individuals in collaboratively exploring and utilizing absorbed knowledge to enhance sustainable innovation remains insufficiently understood [8,29,30]. More specifically, there is a lack of empirical insights into how individuals’ knowledge absorption within collaborative settings shapes the sustainable innovation process in technology-based contexts [31–33].

To address this gap, the present study investigates the following research question: *How does individuals’ collaborative knowledge absorption influence sustainable innovation in technology-driven contexts?* The study integrates effectuation theory and absorptive capacity [34,35,36,37] to conceptualize the framework for the sustainable innovation process under conditions of uncertainty. Effectuation is particularly well-suited to this purpose, as its core principles, such as flexibility, stakeholder collaboration, and iterative experimentation, closely align with the dynamic and uncertain nature of sustainable innovation [5,6]. Moreover, its emphasis on leveraging available means, including stakeholder knowledge and learning, directly corresponds to the mechanisms underlying sustainability-oriented innovation [4].

Empirically, the study examines a case of the development of a hybrid marine engine – a propulsion system for vessels that combines a conventional combustion engine with electric propulsion from batteries – which is a technology-intensive innovation characterized by high uncertainty and strong sustainability ambitions. This case enables an in-depth analysis of the micro-processes through which diverse stakeholders absorb, exchange, and leverage knowledge to navigate uncertainty and translate sustainable innovation from conception to realization. Through the effectuation lens, the study advances an understanding of entrepreneurial behavior, decision-making, and knowledge utilization in sustainability-driven innovation. By identifying distinct phases of the sustainable innovation process, the study elucidates how collaborative knowledge absorption contributes to successful outcomes. Ultimately, the findings provide a structured framework for the strategic allocation and deployment of knowledge resources, contributing to both theory and practice in fostering sustainable innovation in technology-intensive environments.

The remainder of this paper is organized as follows. Section 2 reviews the relevant literature and develops a theoretical framework for sustainable innovation grounded in effectuation and knowledge absorption literature. Section 3 describes the research methodology, while Section 4 presents the results from the case study on the development of a hybrid marine engine, which are used to refine the proposed framework. Sections 5 and 6 discuss the results, identify key phases of the sustainable innovation process, and outline the study’s contributions, limitations, and directions for future research.

## 2 Literature review

### 2.1 A framework for the sustainable innovation process

Sustainability has become a critical consideration and a source of competitive advantage in firms’ innovation activities [38,20], particularly in contexts defined by rapid technological advancements, digitalization, and increasing environmental concerns [39,40]. As public awareness of ecological challenges intensifies, understanding sustainable innovation as a dynamic and evolving process has attracted increasing attention from scholars, practitioners, and policymakers [2,7]. The phenomenon is inherently multifaceted, collaborative, and complex [6,28,26,27], requiring coordinated engagement of diverse actors and resources to co-create social, environmental, and economic value [8,28,27]. Moreover, the process is fundamentally non-linear, unfolding through iterative cycles of opportunity discovery and exploitation [41,42]. To conceptualize this complexity, this study draws on effectuation theory and absorptive capacity as complementary theoretical lenses [34,35,36,37]. It focuses on the processual and micro-level dynamics through which sustainable innovation is enacted, theorizing the underlying mechanisms, individual actions, and collaborative knowledge processes that shape its progression [23–25]. Effectuation theory, which emphasizes navigating uncertainty by leveraging evolving means and emerging opportunities rather than focusing on resource constraints [43], provides a robust foundation for understanding sustainable innovation as an emergent, path-dependent process. By underscoring the utilization of obtainable resources and the strategic accommodation of contingencies [34], effectuation captures the non-linear and emergent nature of the sustainable innovation process [44,45].

### 2.2 Phases of the sustainable innovation process

From an effectuation perspective, the sustainable innovation process originates with stakeholders drawing on obtainable resources – addressing questions such as “Who am I?” (identity resources), “What do I know?” (knowledge resources), and “Whom do I know?” (network resources) – to navigate uncertainty and formulate entrepreneurial goals [34,35]. As the process unfolds, stakeholder interactions and commitments generate new means and new goals in an iterative fashion [34]. Throughout these phases, knowledge absorption plays a crucial role by enabling individuals to acquire, assimilate, transform, and exploit internal and external knowledge [29,36,46], thereby enhancing the effectiveness of the sustainable innovation process [8]. The process thus consists of iterative cycles involving identifying obtainable resources, setting entrepreneurial goals, interacting with other stakeholders, obtaining partnership commitment, and developing new means and new goals [34,35,36,37], continuing until sustainable innovation outcomes are realized. The following subsections elaborate on each phase in detail.

#### Phase 1. Identifying obtainable resources.

The sustainable innovation process begins with the identification and utilization of obtainable resources [34], which serves as a fundamental input for value creation [47], technological advancement [48], and sustainable innovation [12]. These resources can be categorized into three interrelated types: identity, knowledge, and network resources [35,49] – each shaping the distinctive capabilities of individual actors [50] and contributing to the sustainable innovation process.

Identity resources encompass self-awareness, self-confidence, and motivation, all of which influence entrepreneurial decision-making and behavior [51]. These resources shape how entrepreneurs select and deploy other resources, thereby affecting technological innovation trajectories [8,52]. Knowledge resources refer to career-relevant skills and job-related expertise, including both soft and hard skills acquired over time [4]. In a knowledge-based economy, such resources constitute critical strategic assets [53], facilitating competitive advantage and core competencies [8,48]. Network resources, representing an individual’s social capital, include interpersonal relationships and reputational assets that facilitate value creation [4] and promote sustainable innovation [54]. These resources enable access to business opportunities, support continuous learning, and expand diverse professional networks [55]. Importantly, obtainable resources shape entrepreneurial aspiration, which represents the desired goal or outcome of the sustainable innovation process [56]. These aspirations, in turn, drive the translation of obtainable resources into concrete entrepreneurial goals [8,34], influencing the subsequent trajectory of the innovation process.

#### Phase 2. Setting entrepreneurial goals.

Entrepreneurial aspirations constitute the ultimate goals guiding the sustainable innovation process and represent its second phase [34]. These goals evolve alongside obtainable resources [35] and can be categorized into two types: proactive aspirations, which guide actions and enhance knowledge absorptive capacity [57], and reactive aspirations, which involve evaluating prior outcomes to inform future actions [58,59]. Entrepreneurial goals are shaped by individual motivations, personal values, intentions, and risk perceptions [60], which are themselves influenced by education and training [61]. Higher aspirations increase the likelihood of opportunity identification [37]. Moreover, entrepreneurial intentions and personal innovativeness in technology are interconnected [62], a relationship further supported by research highlighting the role of cognition in fostering technology-driven innovation and facilitating collaborative interaction [63], thereby advancing the sustainable innovation process.

#### Phase 3. Interacting with other stakeholders.

Interaction with relevant stakeholders constitutes the third phase in the sustainable innovation process [34,64], enabling a transition from self-reliance to strategic partnerships [65]. As a core principle of effectuation, stakeholder interaction fosters innovation by facilitating idea-sharing, knowledge exchange, knowledge creation, and knowledge absorption [8,66], as well as fostering collaborative opportunities [67]. Technological advancements and increasing knowledge mobility create diverse collaboration possibilities and commitments [48], enriching the sustainable innovation process. Through stakeholder engagement, the pool of obtainable resources expands, and new insights emerge [64,68], making collaboration and commitment essential to the success of sustainable innovation [69]. Effective stakeholder interaction thus propels the process forward.

#### Phase 4. Obtaining partnership commitment.

Commitment represents the fourth phase of the sustainable innovation process [34]. Through interaction and negotiation, founders convince stakeholders of collaboration and commitment to co-create innovations [70]. Strategic alliances further enhance collaboration [35], broadening access to resources and generating new insights [8,68]. In the context of sustainable innovation, rapid technological development necessitates that entrepreneurs cultivate adaptability and accessibility capabilities to respond effectively to evolving market demands [71]. The logic of control – central to effectuation – enables entrepreneurial actors to leverage contingencies as a source of learning and novelty [72]. Commitment and collaboration among stakeholders foster an innovative environment by enabling the sharing of ideas, knowledge, expertise, and opportunities [48,67]. This collaborative approach helps individuals maintain a positive attitude in the face of contingencies and stakeholder diversity [8], thereby enhancing opportunity recognition [73] and advancing the sustainable innovation toward the generation of new means and new goals [34].

#### Phase 5. Developing new means and new goals.

The emergence of new means and new goals marks the fifth phase in the sustainable innovation process [34]. The commitment and collaboration in the previous phase generate increased knowledge mobility [65], providing new options and opportunities for innovation under evolving conditions [74]. These interactions shape a shared vision of the future and culminate in the realization of new means and goals [70]. To navigate this dynamic process, entrepreneurs must develop adaptable strategies that accommodate changing circumstances [71]. Whereas technology was once considered an external factor in innovation, it now plays a central role in product development and value creation [8]. Sustainable innovation thus represents a fundamental shift from a self-reliant, technology-driven approach to a collaborative, network-based paradigm [65].

At the conclusion of the sustainable innovation process, new means and goals emerge through the integration of internal and external resources [34]. This gives rise to feedback loops in which the resource pool expands continually while goals become progressively more refined and constrained [8,34]. These dynamic interactions constitute a critical yet often overlooked dimension of sustainable innovation [75]. Rather than focusing on these broader systemic dynamics, however, the present study specifically examines the role of individuals’ knowledge absorption throughout the process [29].

### 2.3 Knowledge absorption in the sustainable innovation process

The sustainable innovation process is inherently dynamic, with both resources and goals continuously evolving over time [34]. Given the involvement of multiple stakeholders and the presence of uncertainty [70], this study posits that stakeholders’ knowledge absorption is a key driver of this process [36,53]. Knowledge absorption – comprising acquisition, assimilation, transformation, and exploitation – plays a crucial role in problem-solving, value creation, and innovation [48,69,76]. It enables the continuous expansion of resource availability while simultaneously refining and delimiting entrepreneurial goals [48,77–79].

#### Knowledge acquisition in the sustainable innovation process.

Knowledge acquisition represents a foundational component of knowledge absorption in the sustainable innovation process [5,7,29]. It encompasses the recognition, acquisition, and application of new knowledge [37], enabling the identification of potential resources and the recombination of existing knowledge to seize emerging opportunities [80]. This capability is particularly critical in dynamic environments that demand continuous learning and adaptability [59]. Prior knowledge and experience significantly shape an individual’s ability to recognize and acquire new knowledge, influencing future opportunity identification [ 8,79] as well as exploratory learning and network development [81,82]. Specifically, prior related knowledge – including both foundational skills and recent technological advancements – enhances recognition and acquisition processes [83]. The ability to acquire technological knowledge, supported by prior learning [8], facilitates the identification of external knowledge sources [29], reshapes individuals’ knowledge bases [81], and strengthens entrepreneurial aspirations and global competitiveness [84]. Moreover, this capability supports opportunities recognition [12,85] and fosters innovation [48,86], serving as a prerequisite for the subsequent phase of knowledge absorption: knowledge assimilation [5,7,29].

#### Knowledge assimilation in the sustainable innovation process.

Knowledge assimilation constitutes a critical phase of knowledge absorption in sustainable innovation [5,7]. It involves the comprehension and internalization of externally acquired knowledge [81], allowing individuals and organizations to structure and integrate new insights effectively [29]. In the context of sustainable innovation, knowledge sharing among individuals enhances assimilation capabilities [87], influencing how external knowledge is interpreted and utilized [88]. Effective assimilation is facilitated by access to diverse resources, which promotes creativity in the face of uncertainty [89]. Moreover, assimilation capabilities enable entrepreneurial actors to refine and contextualize knowledge, thereby enhancing their responsiveness to environmental and technological change [81]. This process directly supports the subsequent stage of knowledge transformation, further advancing the sustainable innovation process [5,48].

#### Knowledge transformation in the sustainable innovation process.

Knowledge transformation is another essential component of the knowledge absorption, serving as a bridge between exploratory and exploitative learning [81]. It entails retaining, reactivating, and reconfiguring knowledge, thereby facilitating its integration into structured decision-making frameworks [5]. This process involves formalizing and routinizing actions through established rules, procedures, and policies [90]. Additionally, it fosters the development of shared ideologies and broad tacit guidelines that facilitate organizational learning [57]. Prior technological and market knowledge enhances the flexibility required to adapt to evolving environmental conditions [91,92]. Effective knowledge transformation is integral to sustainable innovation, as it enables entrepreneurs to leverage contextual insights and convert abstract ideas into actionable strategies [10,32]. Moreover, transformation strengthens decision-making relationships and improves the overall efficiency of knowledge absorption [90]. In environments characterized by continuous learning and knowledge adaptation, the potential for technological innovation is significantly enhanced [6,48], setting the stage for the final dimension of knowledge absorption: knowledge exploitation [5,7,29].

#### Knowledge exploitation in the sustainable innovation process.

Knowledge exploitation represents the final dimension of knowledge absorption, focusing on the integration and application of acquired, assimilated, and transformed knowledge within the sustainable innovation process [5]. The mere accumulation of knowledge does not inherently lead to innovation; rather, innovation arises from the ability to combine, contextualize, and effectively apply knowledge to real-world challenges [90]. The exploitation process enables sustainable innovation by facilitating the application of existing knowledge to novel contexts and technological advancements [5]. It involves both the refinement of established knowledge and the exploration of untapped technological potential, often yielding breakthrough innovations through incremental learning and recombination [48].

Ultimately, the four dimensions of knowledge absorption – acquisition, assimilation, transformation, and exploitation – function as an interconnected system that drives the sustainable innovation process [7,29,48]. By fostering continuous learning and strategic knowledge management, these capabilities enhance an organization’s ability to navigate technological complexities, respond to dynamic market conditions, and achieve long-term sustainability.

### 2.4 Literature-based framework

**Fig 1:**
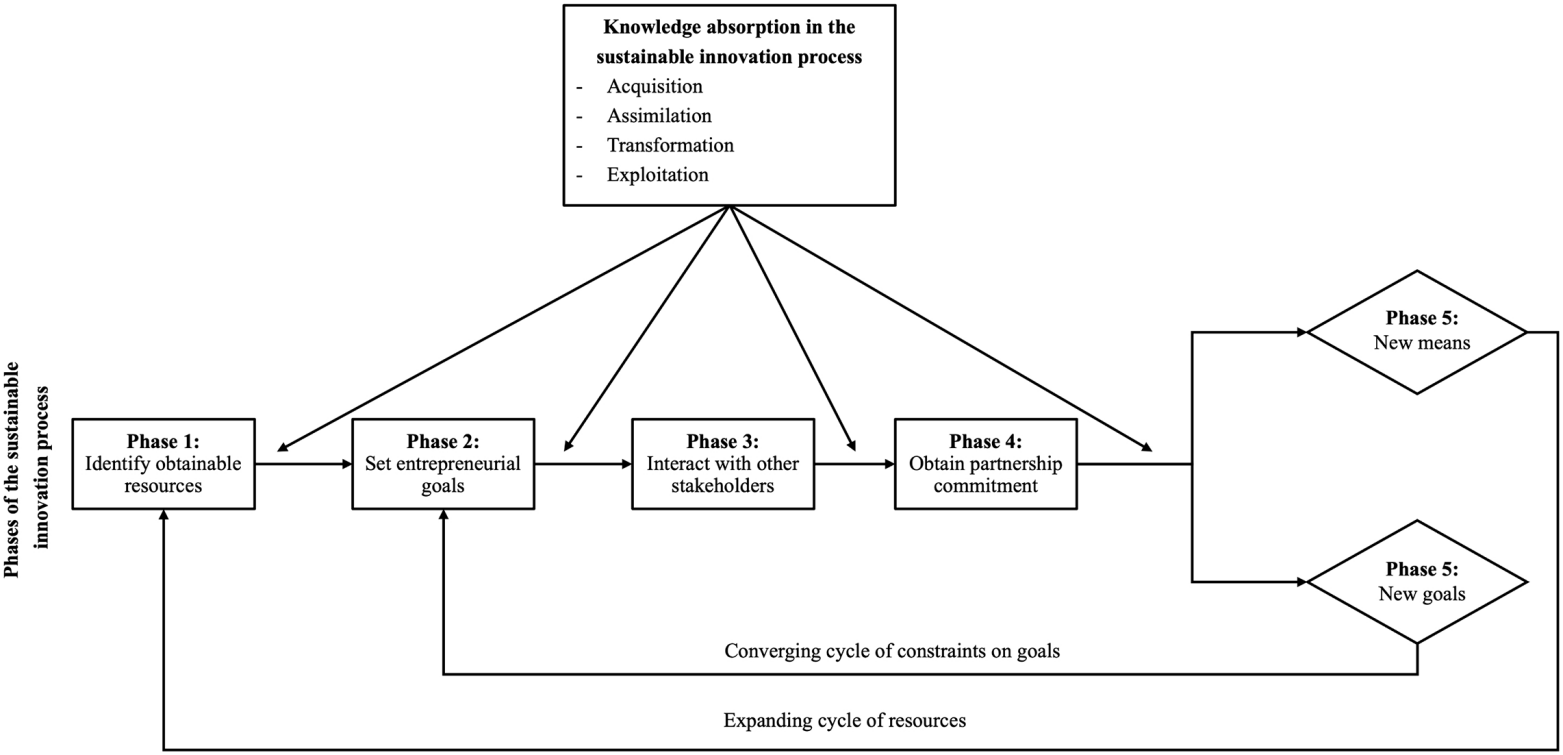
Literature-based framework of the sustainable innovation process integrating effectuation and knowledge absorption.

Based on the literature review, we propose a literature-based framework for the sustainable innovation process consisting of five phases, driven by four aspects of stakeholders’ knowledge absorption, with feedback loops from the last phase to the first two phases of the sustainable innovation process (see Fig 1).

## 3 Methodology and methods

### 3.1 Selection of the empirical case study

This study adopts an abductive, iterative qualitative research methodology to examine the role of stakeholders’ knowledge absorption in the sustainable innovation process. An initial framework for the sustainable innovation process was developed by integrating effectuation and absorptive capacity theory [34,35,36,37]. This framework is derived from a comprehensive review of the existing literature and subsequently iteratively refined through empirical insights generated during the case study [93,94]. Through this iterative theory-data interplay, the analysis resulted in a refined literature-based framework that is grounded in case-study based empirical evidence [95,96], thereby constituting a robust conceptual contribution [97].

The empirical case study focuses on the development of a hybrid marine engine, which is a sustainable innovation process involving multiple collaborating stakeholders. Each stakeholder contributes to the innovation process by leveraging its knowledge-absorption capabilities to advance high-technology innovation. By integrating theoretical perspectives of effectuation and knowledge absorption theory with empirical findings, the evolving framework demonstrates explanatory power and analytical validity for comparable technological-driven cases and contexts, in line with case study methodology literature [98,95].

### 3.2 Data collection methods and process

**Fig 2:**
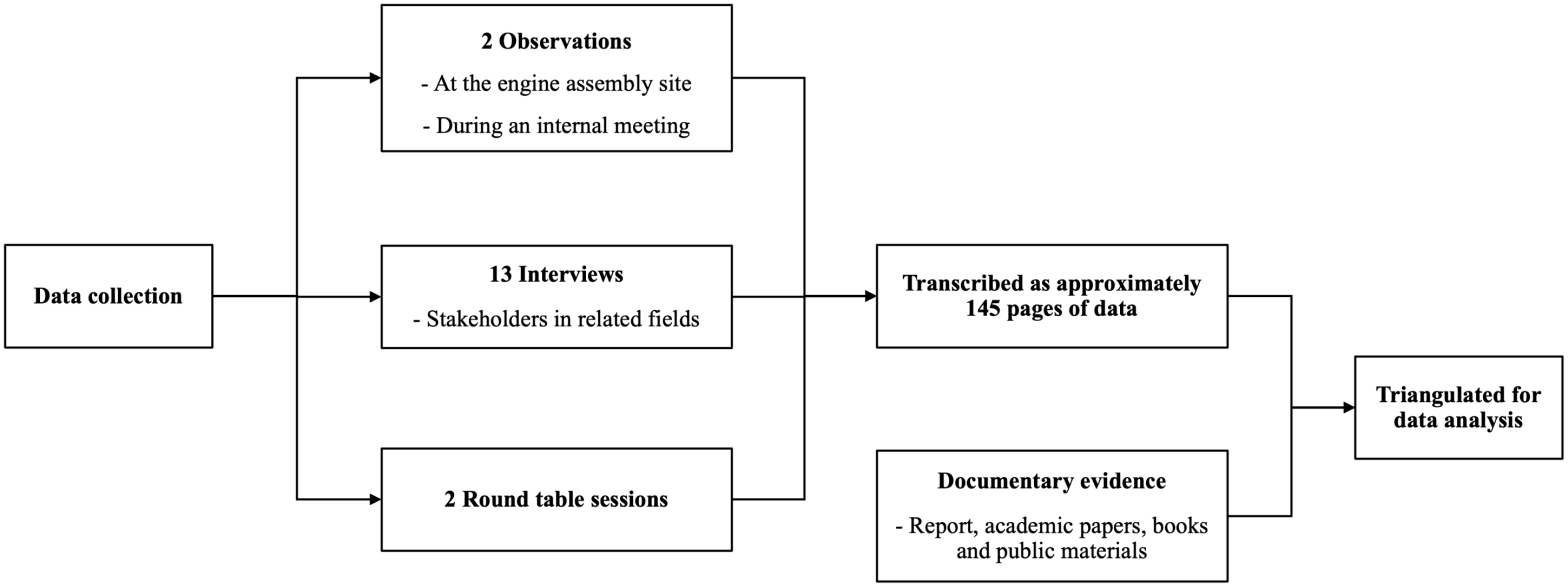
Data collection process.

Multiple data collection methods were employed, including semi-structured interviews, observations, roundtable sessions, internal documents, and publicly available documents (see Fig 2). Data triangulation was used to minimize sample selection bias and to enhance the internal and analytical validity of the findings [93,98,95]. Specifically, primary data were collected through observations, semi-structured interviews, and roundtable sessions conducted via Zoom meetings between February and May 2021. In addition, an extensive search for documentary evidence was undertaken, including relevant reports, academic publications, and publicly available materials related to the development of a hybrid marine engine. In accordance with the university’s Research Data and Software Management Policy, the data have been stored on the university’s secure, authorized server and are available upon reasonable request.

### 3.3 Characteristics of the development of a hybrid marine engine case study

The empirical setting of this case study comprises a collaborating network of stakeholders engaged in the development of a hybrid marine engine in the Netherlands, a case of sustainable innovation within a technology-intensive, multi-stakeholder context. Rather than focusing on a single company, the case involves several interrelated organizations and individuals jointly involved in the research, development, and commercialization of hybrid propulsion technology.

The stakeholders participating in this process include business owners, engineers, developers, suppliers, distributors, clients, and environmental officers from non-governmental organizations (NGOs), all of whom either directly contribute to or influence the innovation trajectory of the hybrid marine engine. This configuration reflects the distributed and systemic nature of sustainable innovation, which typically transcends organizational boundaries and depends on the collaborative absorption and integration of knowledge across institutional, organizational, and disciplinary domains.

The hybrid marine engine project provides a particularly fitting empirical context, as it involves multiple firms and institutions operating across different stages of technological development, ranging from R&D laboratories and component manufacturers to marine engineering firms, environmental NGOs, and clients adopting the innovation. This diversity enabled an in-depth examination of the cross-organizational learning, knowledge integration, and decision-making dynamics that shape sustainable innovation processes and outcomes.

To ensure alignment with the research objective and research question, the study adopted a theoretically informed case selection strategy [94,95], which prioritizes the selection of cases based on their potential to contribute to the development, refinement, or extension of theoretical concepts rather than statistical representativeness [95,96]. In this study, the case and its participants were deliberately selected because they embody the inter-organizational collaboration mechanisms central to understanding how knowledge absorption and co-creation unfold in sustainable innovation within a technology-intensive setting. Thus, the case was chosen for its heuristic and revelatory potential to illuminate the collaborative processes that enable the development of complex, sustainable technologies.

Table 1 provides an overview of the participating organizations and their respective roles in the hybrid marine engine development process. Together, these entities form a representative ecosystem of actors jointly propelling this sustainable technological innovation toward market realization. Due to the confidential nature of the technology under development and the anonymity agreements associated with the interviews, detailed company-level identifiers (e.g., names, sizes, or ownership structures) are not disclosed.

**Table 1:** Summary of stakeholders included.

Stakeholder type	Number of participants	Method of data collection
Distributors and developers	4	Zoom online Interview (30 mins -1h)
Academia stakeholders	2	
Clients	2	
Environmental NGOs	2	
Infrastructure provider	1	
Technology expert	1	
Naval architect	1	

Insights from the extensive search for documentary evidence informed the development of the interview protocol for primary data collection, which also included guidelines for obtaining participant consent, ensuring privacy, outlining interview procedures, and detailing data collection and analysis methodologies. The interviews, each lasting between 30 and 60 minutes, provided detailed insights into specific situations, activities, and decision-making processes. Additionally, two observations were conducted: one in March 2021, at the engine assembly site, involving eight entrepreneurial individuals, including the CEO, actively engaged in the engineering process and interactions with other stakeholders; and another in April 2021, at a meeting where the engine operations manager discussed offering comprehensive solutions to end-users. Detailed field notes were taken during these observations and promptly organized thereafter.

### 3.4 Ethics statement

The authors confirm that this research adhered to ethical standards and guidelines throughout the research process. The study followed ethical protocols in accordance with institutional and international guidelines, including maintaining respondent confidentiality and anonymity, using collected data exclusively for the purposes outlined in the study, ensuring transparency in data handling and reporting, and avoiding any form of coercion or undue influence in securing participation.

The authors declare that:

1 *Ethics review.* The research ethics self-check conducted by the Ethics Review Committee of the Faculty of Science (BETHCIE), Vrije Universiteit Amsterdam, confirmed that the research project did not require further evaluation by the Research Ethics Review Committee.
2 *Participant inclusion and consent.* Prior to data collection, participants received emails detailing the study’s objectives, their right to decline participation, and their ability to withdraw at any time. The authors affirm that participants’ autonomy and confidentiality were rigorously upheld and respected throughout the study, that participants were fully informed about the research purpose, their rights as respondents, and the measures implemented to ensure their confidentiality and anonymity. Additionally, they were explicitly given the option to withdraw from the study at any stage.
3 *Informed consent.* No formal informed consent was obtained from respondents, and all data were analyzed anonymously. No personally identifiable information was recorded. The consent was audio-recorded during the interview, which BETHCIE approved.
4 *Participant criteria.* No minors were included in the study.

### 3.5 Data analysis method

**Fig 3:**
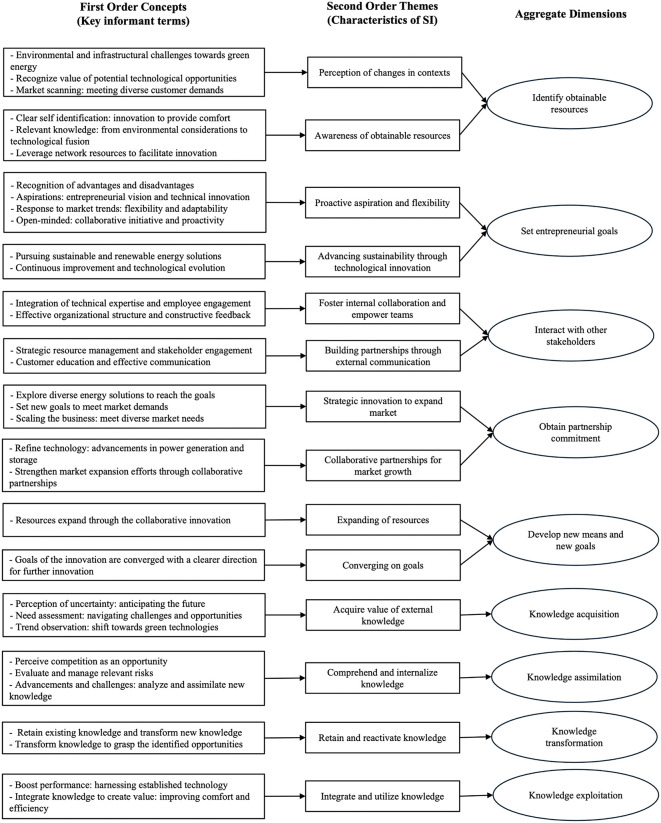
Data structure developed using methodology developed by Gioia et al.

All interview recordings were transcribed verbatim and imported into Atlas.ti for systematic analysis following the methodology developed by Gioia et al. [97], in which data were coded and sensitized to literature-based concepts. The coding procedure followed an open and axial coding process [97]. The open coding process began with labeling the gathered and recorded data, while the axial coding process seeking similarities between these labels to create second-order categories [97]. These categories were then compared and allocated across the capacities of knowledge absorption (acquisition, assimilation, transformation, and exploitation) and their proposed impact on the phases of the sustainable innovation process, as described in the literature section and visualized in Fig 1. The results of this coding process, which includes labeling, grouping key informant terms into second-order categories, and sensitizing these to literature-based dimensions [97], are presented visually in Fig 3.

### 3.6 Synthesis of literature-based and empirical findings into a framework

**Fig 4:**
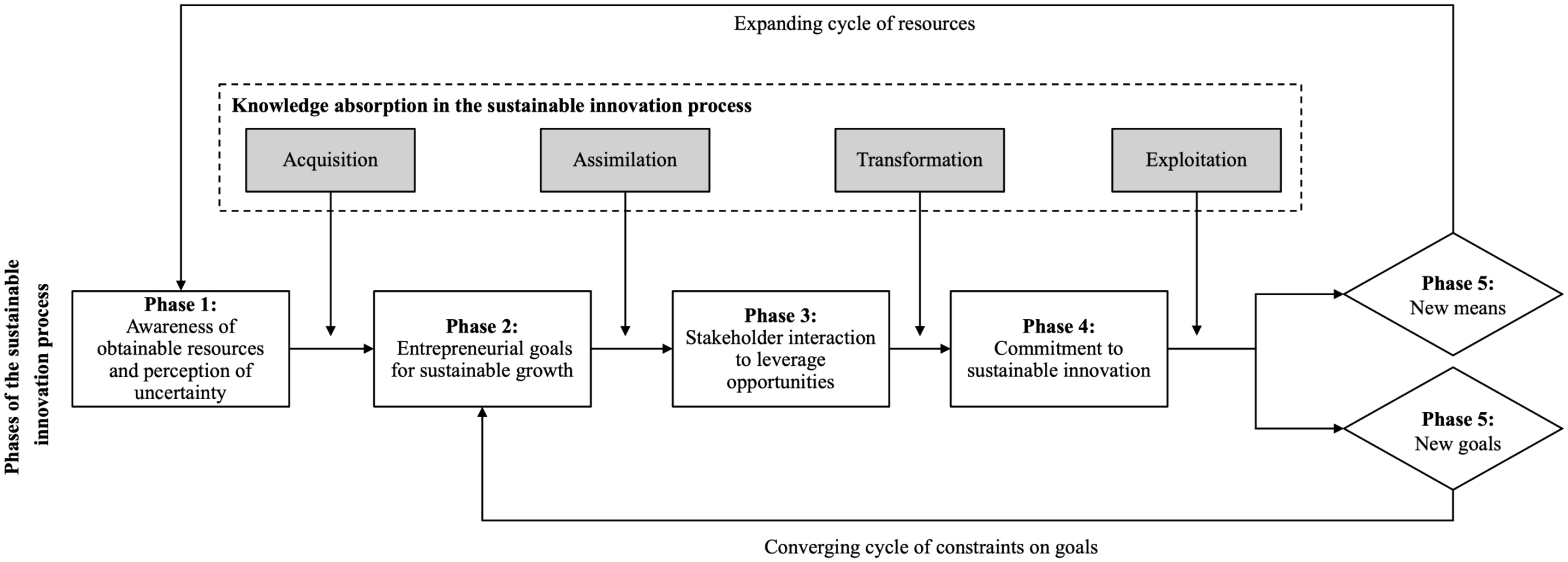
Literature- and case study-based sustainable innovation framework.

The iterative interplay between theory and data resulted in a final framework through several steps. First, a preliminary codebook was developed based on the initial theoretical framework, which included the phases of resources, goals, interactions, commitment, and new means and goals. Second, after familiarizing with the data, the initial codes were applied. However, the analysis revealed the need to refine it to more accurately reflect the case’s empirical nuances. For example, the initial code ‘obtainable resources’ was divided into two distinct themes: ‘Perception of changes in contexts’ and ‘Awareness of obtainable resources’, to better represent participants’ emphasis on resource accessibility amid uncertainty (See Fig 3 for the full data structure). The refined codebook was subsequently applied to the entire dataset. During this process, additional emergent concepts and patterns were documented through analytic memos. Third, through iterative comparison and reflection, overlapping or ambiguous codes were consolidated, and the relationships among categories were re-examined to ensure both conceptual coherence and empirical grounding. Finally, the coding structure was sensitized to the literature-based concepts and synthesized into a refined framework representing the stages of the sustainable innovation process (See Fig 4). To ensure the reliability and validity of the findings, a second researcher independently reviewed a subset of coded data. Differences in interpretation were discussed until consensus was achieved, strengthening the rigor and consistency of the analytical process.

## 4 Results

### 4.1 Literature- and case study-based sustainable innovation framework

This section presents the empirical findings and insights derived from an in-depth case study of the development of a hybrid marine engine. Consistent with a theory-driven approach to case study research [95], the analysis builds on an initial conceptual framework developed from prior literature (see Section 3 and Fig 1). Guided by abductive principles [96] and following the methodology developed by Gioia et al. [97], this framework is iteratively confronted with the empirical material, leading to the data structure presented in Fig 3, and to developing the literature- and case study-based framework shown in Fig 4.

The empirical analysis identifies five interrelated phases of the sustainable innovation process, each characterized by specific attributes. As shown in the data structure (Fig 3), the four dimensions of knowledge absorption, including acquisition, assimilation, transformation, and exploitation of knowledge, play a moderating role across different phases of the process. These phases encompass (1) awareness of obtainable resources and perception of uncertainty, (2) entrepreneurial goals for sustainable growth, (3) stakeholder interaction to leverage opportunities, (4) commitment to sustainable innovation, and (5) emergence of new means and goals. These phases are not discrete steps but interconnected mechanisms through which stakeholders interpret uncertainty, absorb knowledge, and reconfigure relationships to achieve sustainability-oriented innovation.

Phase 1 awareness of obtainable resources and perception of uncertainty function as triggering mechanisms that convert uncertainty into opportunity through effectual reasoning and absorptive capacity, thereby advancing technological innovation toward achieving entrepreneurial goals for sustainable growth. Phase 2 serves as the transitional point that enables firms to value and internalize external knowledge for strategic direction and links the awareness of obtainable resources to transition the sustainable innovation process to the next phase. Phase 3 stakeholder interaction to leverage opportunities demonstrates how effectual networks transform entrepreneurial intentions into experimentation, emphasizing the significance of collaboration in the sustainable innovation process. It fosters collective interpretations, rather than top-down coordination, therefore driving innovation trajectories. Such collaborative efforts enable all stakeholders in the sustainable innovation process to contribute their expertise and foster a commitment to sustainable innovation, ultimately translating identified opportunities into technological advancements. Phase 4 commitment to sustainable innovation involves a shift from exploratory flexibility to design routines, partnerships, and commercialization strategies, operating as a conversion point where entrepreneurial goals are not aspirational but materially embedded in product and process development. It demonstrates how absorptive capacity is operationalized in the innovation process, ensuring that all relevant stakeholders benefit and enhance the technology innovation process to achieve new means and goals. Phase 5 emergence of new means and goals highlights the learning accumulation mechanism, in which outcomes from prior action become new means for future experimentation, reinforcing the iterative nature of effectuation under uncertainty, where sustainable innovation remains an open-ended, evolutionary process rather than a finite achievement.

Further analysis reveals that specific aspects of knowledge absorption among collaborating professionals exert a dominant influence on transitions between phases and on internal dynamics within each phase. These findings extend existing studies on absorptive capacity and effectuation by showing that sustainable innovation unfolds through recursive cycles of learning, interaction, and adaptation rather than through predetermined sequences. Moreover, the empirical insights enabled a more detailed specification of the sustainable innovation process, which is reflected in the literature- and case study-based framework presented in Fig 4. These phase-specific dynamics and mechanisms are elaborated in the following subsections, supported by representative quotations that further substantiate the analytical claims.

### 4.2 Empirical case of sustainable innovation: the development of a hybrid marine engine

The concept of a hybrid system, invented initially over a century ago and once used in submarines, has now been adopted for marine engines to address evolving market demands. Unlike a standalone product, a hybrid marine engine is a complex technical system that requires the involvement of multiple stakeholders, including infrastructure providers, developers, distributors, technology experts, environmental stakeholders, and end users. The development of a hybrid marine engine extends beyond conventional product innovation. It represents a collaborative process that integrates diverse stakeholders to achieve sustainability. This process involves key stakeholders, including internal employees, external suppliers, customers, and competitors, as well as other relevant stakeholders, thereby fostering a dynamic ecosystem of knowledge exchange and co-creation. By strategically combining the advantages of diesel, electric propulsion, and other clean energy sources, this collaborative approach drives technological advancements and enhances sustainability within the innovation process.

### 4.3 Phases of the sustainable innovation process based on the empirical case

Five distinct phases of sustainable innovation are observed in the case on the development of a hybrid marine engine, including (1) awareness of obtainable resources and perception of uncertainty, (2) entrepreneurial goals for sustainable growth, (3) stakeholder interaction to leverage opportunities, (4) commitment to sustainable innovation, and (5) emergence of new means and goals. Across all these phases, the logic of effectuation explains how stakeholders act under uncertainty, and the framework of absorptive capacity clarifies how the flow of knowledge enables these transitions. Sustainable innovation can be depicted as an iterative sequence characterized by continuous knowledge recombination and goal redefinition. Below is a description of the phases as found in the case study, interspersed with a selection of illustrative quotes from interviews. Appendix A provides a complete overview of remaining relevant interview quotes per phase and of the four aspects of knowledge absorption, which were not integrated in the main case descriptions for reasons of conciseness.

#### Phase 1. Awareness of obtainable resources and perception of uncertainty.

The initiation of the development of a hybrid marine engine is marked by an awareness of obtainable resources and a perception of uncertainty. At this early stage, individuals interpret external uncertainties such as environmental pressures, technological advancement, shifting customer preferences, social challenges, policy incentives, and navigate these complexities. Empirically, interviewees highlighted government grants as triggers for re-evaluating existing resources. As S2 noted:

“*There is currently a governmental grant for fishermen to employ engines that consume 30% less. That is very achievable with hybrid engines (...)*”

Additionally, policy initiatives support alternative fuel sources, as highlighted by interviewee S2:

*“There was one call for action by the fund for environment preservation, for funding vessels on alternative fuel sources. That was for electricity and hydrogen.”*

Moreover, market trends also act as a driver of sustainable innovation, as emphasized by interviewee S5:

*“The main reason was the client’s demand.”*

and interviewee S3 added:

*“Depending on the market trend, the concept can be developed with different sources and directions.”*

Rather than passively responding to uncertainty, stakeholders engaged in effectual reasoning to redefine constraints as starting points for innovation through asking key questions regarding their identity, knowledge, and networks: “Who am I?” (identity resources), “What do I know?” (knowledge resources), and “Whom do I know?” (network resources). This cognitive reframing transforms environmental uncertainty into a domain of opportunity. For example, as one developer interviewee S7 explained:

*“You want to be in a new market, and the new market has new specific requirements that you follow with technicalities.”*

Technology feasibility is another significant consideration, as noted by interviewee S6:

*“The rising demand is for hybrids because it is a technical realism. It is a transient realism.”*

By leveraging their expertise in diesel engine technology and boat construction, developers identify technological opportunities through market analysis and trend observation. As interviewee S6 elaborated:

*“The first step we are witnessing today is this hybrid system, between electric engines and their performances, and engines which give great autonomy like diesel engines. This fusion brings us to the hybrid. That is how we started developing hybrids.”*

The mechanism underlying this transition is a means-driven principle. Stakeholders draw on prior technical and relational knowledge to construct plausible paths forward, aligning with the initial step of the effectuation process, where the sustainable innovation process starts with controllable means rather than predictive goals. Meanwhile, early scanning and evaluation of technologies indicate the potential knowledge absorptive capacity.

#### Phase 2. Entrepreneurial goals for sustainable growth.

The second phase of the development of a hybrid marine engine is establishing entrepreneurial goals for sustainable growth. Once stakeholders recognize the potential of hybrid technology and related uncertainties, they begin to define entrepreneurial goals that balance sustainability ambitions with market and technological realities. This aligns with the effective principle of flexibility and adaptability, where adaptive objectives rather than fixed targets are set to navigate internal and external contingencies, fostering innovation. As interviewee S5 observed:

*“A micro company needs to be flexible in its strategy and susceptible to market fluctuations. It cannot be rigid. This is also an advantage because a micro company can adapt fast.”*

This flexibility enables developers to effectively assess different propulsion options, respond to customer demands, and align their aspirations with environmental regulations. Forward-looking strategies explore renewable energy integration to achieve sustainability, as interviewee S11 noted:

*“In the future, we need to find a way to utilize the energy from wind or solar and to put this energy into the propulsion for the ships.”*

Within this context, entrepreneurial aspirations also emphasize enhancing user experience and comfort. As stated by interviewee S3:

*“We try to sell more comfort and convenience. The electric propulsion system in the hybrid system is the next level of comfort.”*

By evaluating market demand, competitive landscapes, technological feasibility, and associated risks, entrepreneurial goals for sustainable growth act as critical drivers in fostering stakeholder interactions to leverage opportunities. Moreover, entrepreneurial goals show how stakeholders absorb knowledge, developing goals that bridge technological feasibility and market desirability.

#### Phase 3. Stakeholder interaction to leverage opportunities.

The third phase of the development of a hybrid marine engine entails stakeholder interaction to leverage opportunities. Here, innovation progresses from individual intention to the collective realization of opportunity. The development of a hybrid marine engine extends beyond product creation, requiring engagement with internal employees, external suppliers, customers, and competitors. Stakeholders engage in reciprocal exchanges that allow the pooling of knowledge, resources, and legitimacy. For example, market insights and customer feedback play a critical role, as interviewee S5 explained:

*“(…), if there is a need for mutual reinforcement and leveraging inter-organizational relationships, as a director, I support every initiative. However, this has to be mutual.”*

Collaboration with suppliers, customers, and competitors is crucial for optimizing resources and enhancing efficiency. As interviewee S7 highlighted:

*“If we bought a machine for material processing, we would use it maybe for ten days per year. That would be the same scenario for a honing machine. That doesn’t make sense, so that’s why we outsource it.”*

Customer co-creation further enhances stakeholder engagement, as interviewee S5 emphasized:

*“For me, the client is someone who supports the creation of value and someone who invests in it.”*

#### Phase 4. Commitment to sustainable innovation.

In the fourth phase, stakeholders demonstrate commitment to sustainable innovation by integrating advanced technologies to create value. Commitment functions as the stabilization mechanism that converts exploratory collaboration into enduring innovation pathways. Empirically, stakeholders emphasize both technological and environmental motivations. As Interviewee S3 observed:

*“When you stop, the hybrid is silent. No noise, no vibration, while the solar energy can possibly recharge the batteries.”*

Additionally, Interviewee S4 also noted

*“I think that currently hybrid solutions are the best solution for their applications because they balance different sources of energy and also reduce exhaust emissions.”*

Drawing inspiration from electric trains and cars, developers optimize marine vessels for efficiency while addressing challenges such as noise, emissions, and space limitations. Long-term collaborations and strategic commercialization ensure continuous improvement and technological advancement.

#### Phase 5. New means and new goals.

The final phase entails the realization of new means and new goals, such as increased resources and market expansion, and further development possibilities to achieve sustainability. It represents the system’s capacity for continuous evolution and demonstrates the outcome of stakeholders’ accumulated knowledge absorption. Collaboration plays a vital role in this progression. As interviewee S7 noted:

*“By broadening the market with our scope, you and we can also work globally through hybrid technology. You are not limited anymore. Suddenly, you have a great expansion.”*

Stakeholder collaboration fosters knowledge integration, enhancing innovation potential. As interviewee S5 explained:

*“By using collaboration with more partners, you can achieve a better result because you accumulate more knowledge.”*

Furthermore, meeting diverse market demands enhances adaptability and customization. As Interviewee S13 stated:

*“For the larger yachts, we are exploring hybrid solutions, so fossil fuel engines in combination with electric propulsion and advanced batteries.”*

This phase reflects the culmination of efforts of all stakeholders, leveraging available opportunities to drive continued technological advancements in the hybrid marine engine to achieve sustainability in the innovation process.

### 4.4 Knowledge absorption in the sustainable innovation process

In the trajectory of the development of a hybrid marine engine, this study identifies the absorptive capacity of collaborating stakeholders as a critical factor in enhancing sustainable innovation. The data reveal that the four dimensions of absorptive capacity —knowledge acquisition, assimilation, transformation, and exploitation —each play a distinct role at different phases of the innovation process, collectively strengthening sustainability. Specifically, knowledge acquisition facilitates the transition from awareness of obtainable resources to the establishment of entrepreneurial goals for sustainable growth. Knowledge assimilation facilitates the transition from entrepreneurial goals to stakeholder engagement, where opportunities are effectively leveraged. Knowledge transformation is instrumental in advancing from stakeholder interaction to a commitment to sustainable innovation, while knowledge exploitation is essential for translating this commitment into the realization of new means and new goals.

#### Knowledge acquisition to transition from phase 1 to phase 2.

Knowledge acquisition is fundamental in transitioning from the awareness of obtainable resources and perception of uncertainty to the establishment of entrepreneurial goals for sustainable growth. Following the identification of obtainable resources, the innovation process requires the continuous acquisition of environmental, technological, and market knowledge from the external contexts to achieve entrepreneurial goals for sustainable growth. As interviewee S2 noted:

*“Every day, we are still acquiring novelties on diesel engines; everything is constantly developing.”*

By responding to evolving customer demands and integrating both explicit and tacit knowledge, developers effectively adapt and deploy resources to meet diverse technological and market requirements. Through this ongoing acquisition, they gain unique insights that shape future innovation, as highlighted by interviewee S11:

*“The changes are happening. From the end user, yes. But, if you ask the end user, (s)he will say (s)he is looking for a more comfortable way of propulsion. The biggest thing is that most people are not aware.”*

Moreover, the accumulation of relevant knowledge strengthens existing expertise, enabling developers to anticipate future trends and improve the innovation process. Interviewee S13 emphasized this forward-looking perspective:

*“Future generations will be more open to clean technologies and to pay for it.”*

Prior knowledge and problem-solving skills further facilitate continuous learning and performance enhancement, fostering innovative associations that can be leveraged for new product development and market adaptation. As interviewee S6 explained:

*“We are now in a transition period to electric propulsion. The source of electric energy is either a nuclear power plant on board or hydrogen, which produces electricity through fuel cells. This is a way to develop energy on board.”*

Thus, knowledge acquisition enables stakeholders to recognize future trends, equipping them to respond proactively to emerging challenges and opportunities in the innovation process. This process is further strengthened through the assimilation of knowledge.

#### Knowledge assimilation to transition from phase 2 to phase 3.

Knowledge assimilation is essential in progressing from setting entrepreneurial goals for sustainable growth to engaging in stakeholder interactions to leverage opportunities. It involves the interpretive integration of newly acquired information into shared cognitive frames that guide coordination. Through assimilation, stakeholders collectively make sense of evolving market dynamics and reconstruct the meaning of competition, uncertainty, and opportunities. Empirically, stakeholders describe competition as a potential partnership rather than a threat. Interviewee S4 exemplified this perspective:

*“I perceive competition as an opportunity because our core business is the distribution of diesel engines. We are one of the main components in a hybrid system. If we have the chance of developing a hybrid system with different suppliers on the market, then it’s an opportunity for us.”*

This assimilation of knowledge empowers stakeholders to integrate newly acquired knowledge into their strategic positioning within the hybrid marine engine sector. As interviewee S4 further noted:

*“We are stimulated by increasing demand, and we are already stakeholders with different electric engine suppliers.”*

Assimilating knowledge also enables stakeholders to more clearly define their role within the evolving ecosystem of the development of a hybrid marine engine. As interviewee S10 stated:

*“We are not engine specialists but sailing specialists.”*

By continuously adapting to the dynamic market landscape, collaborating developers explore new avenues for engagement, shaping industry standards through interaction and collaboration. As interviewee S2 observed:

*“Eventually, with the effort of the operator, i.e., boat owner, this sequence will bring improvements.”*

The assimilation of knowledge fosters confidence in the strategic direction of innovation, aligning stakeholder interactions with entrepreneurial goals that aim to enhance user experience and sustainability. It functions not only as an information integration mechanism but also as a cognitive alignment mechanism among stakeholders. By collectively interpreting market signals, stakeholders reframe competition as a potential for collaboration, consistent with the effectual principle of leveraging contingencies. This process paves the way for knowledge transformation.

#### Knowledge transformation to transition from phase 3 to phase 4.

Knowledge transformation is crucial in advancing the development of a hybrid marine engine from stakeholder interaction and opportunities leveraging to a strengthened commitment to sustainable innovation. In the rapidly evolving market, the ability of founders and developers to convert assimilated knowledge into actionable strategies is essential for fostering innovation. This is particularly evident in the cultivation of long-term client relationships, as highlighted by interviewee S2:

*“By having a complex system, the seamen will have to get acquainted with it. Once a person is acquainted with such a system, (s)he will be able to service it and take care of it more, which is a benefit in the long run.”*

Throughout the innovation process, developers demonstrate effective knowledge transformation by rapidly responding to customer preferences, strengthening both internal and external interaction to drive the sustainable innovation process. Interviewee S5 provided a concrete example:

*“An existing client who consumed a diesel engine wanted to switch to using diesel only half of the time for his drive. That is how the idea and trigger for collaboration were born. If we didn’t consider this, we would probably lose the client. Not listening to the market’s needs, means losing the market.”*

The knowledge transformation capacity fosters an open and improvement-oriented mindset, allowing developers to integrate new insights and enhance their commitment to sustainable innovation. It is the micro-foundations of dynamic capabilities, where stakeholders reconfigure relational and cognitive routines to incorporate new knowledge. Transformation here involves not just adaptation but recombination, the deliberate synthesis of customer input and technological know-how into a novel system. Such recombination demonstrates how absorptive capacity and effectuation intersect, with knowledge transformation acting as a mechanism for converting effectual experimentation into path-dependent learning. This commitment is further reinforced through knowledge exploitation.

#### Knowledge exploitation to transition from phase 4 to phase 5.

Knowledge exploitation is critical in transitioning from a commitment to sustainable innovation toward the realization of new means and goals. The effective integration and application of knowledge drive the advancement of hybrid marine propulsion systems, with an emphasis on optimizing comfort, efficiency, and sustainability. This iterative process enables developers to refine their technological solutions and expand market reach. Interviewee S6 underscored this point:

*“Complex technologies with time…. They require more knowledge from the engineer for engineering and development, but once they have been realized, there isn’t much maintenance afterwards.”*

By exploiting acquired and transformed knowledge, developers not only meet specific market requirements but also expand their innovation potential, fostering broader technological progress. Interviewee S10 highlighted the versatility of hybrid propulsion systems:

*“When you have the diesel running, you could use the electric engine as a shaft generator, but you could also use it for that purpose when you are sailing and use the propeller as a hydro generator. So, there are many different modes of operation.”*

In the sustainable innovation process, developers strategically explore diverse applications of existing knowledge to address evolving customer demands and integrate sustainable energy solutions. Interviewee S13 emphasized the importance of practical validation:

*“We are trying to confirm the performance stated in the design plan. Afterwards, we want to put the dyno to realization.”*

Accumulated expertise enables developers to incorporate novel insights into product development, adapt solutions to shifting market needs, and respond effectively to customer expectations, fostering sustainable innovation. Interviewee S10 elaborated on ongoing research efforts:

*“At the moment, we are looking into different ways of storing hydrogen, liquid or gaseous, under high pressure or low temperature.”*

Therefore, by exploiting knowledge relevant to electric engines and alternative clean energy sources, developers expand their resource base, facilitate technology integration, and foster innovative solutions that create long-term value for customers and achieve sustainability. The empirical patterns reveal that absorptive capacity dimensions collectively operationalize the effectuation process within sustainable innovation. Knowledge acquisition corresponds to the means-driven exploration of obtainable resources, assimilation reflects the co-creation of stakeholders’ networks, transformation manifests as iterative experimentation, and exploitation embodies the convergence toward new goals.

## 5 Discussion

This study develops a process-oriented theoretical framework for sustainable innovation process in technology-driven contexts under uncertainty, drawing on effectuation theory and absorptive capacity [34,99,100]. Rather than extending these theories independently, the study integrates them to explain how collaborative knowledge absorption enables sustainable innovation across organizational boundaries. By applying effectual logics to a sustainability-oriented, multi-stakeholder innovation setting, the findings refine existing interpretation of effectuation and demonstrate the central role of knowledge absorption as a boundary-spanning capability that links cognition, interaction, and action throughout sustainable innovation.

Methodologically, the framework is validated through an in-depth qualitative case study that enables process tracing across phase of innovation. the empirical analysis relies on iterative coding, temporal bracketing, and triangulation across multiple data sources, allowing theoretical constructs to be systematically linked to observed actions and decisions. This approach strengthens internal validity by demonstrating how transitions between phases are empirically grounded in distinct dimensions of knowledge absorption, while supporting analytical generalizability through theory elaboration rather than statistical inference.

### 5.1 Phases and characteristics of the sustainable innovation process

The findings indicate that sustainable innovation unfolds through a set of sequential yet overlapping phases, each characterized by distinct configurations of knowledge absorption and effectual decision-making. These phases integrate cognitive, relational, and operational dimensions of knowledge and collectively form a dynamic, non-linear process. The process begins with awareness of obtainable resources and perception of uncertainty, reflecting the means-driven principle of effectuation and evolving as stakeholders navigate external contingencies [101]. At this phase, stakeholders assess technological, organizational and relational resources while interpreting environmental uncertainty not as constraints, but as stimuli for proactive learning and adaptation. Sustainability-oriented uncertainty – such as regulatory shifts or environmental pressures – triggers early knowledge acquisition and shapes problem framing. This phase establishes the cognitive conditions for innovation readiness.

Following this, the process advances to a phase involving the alignment of entrepreneurial goals, during which collaborating individuals engage in knowledge assimilation to transform perceived challenges into shared opportunities for sustainable value creation. This phase extends the effectual principle of leveraging contingencies by explicitly embedding environmental and social objectives into goal formation. To facilitate sustainable innovation, stakeholders assimilate specific resources and construct opportunity frames through idea generation, knowledge exchange, and the identification of collaborative opportunities, all of which are supported by active stakeholder interaction [8,37,67]. Knowledge assimilation functions as a relational mechanism that enables shared understanding across diverse actors, reconciling short-term market imperatives with long-term sustainability goals.

As interaction intensifies, the process advances to stakeholder interaction to leverage opportunities, where previously perceived competitive threats are cognitively reinterpreted as potential collaboration opportunities. Consistent with effectual co-creation, knowledge assimilation not only enhances knowledge integration but also serves as a cognitive coordination mechanism that aligns stakeholders around emergent opportunities rather than market rivalry. Empirically, participants demonstrated this reframing when distributors and developers perceived competitors as possible partners in hybrid system integration. This extends traditional formulations of effectuation by situating them within network-level, sustainability-driven collaboration [35,62,77]. Such collaborative processes foster continuous learning, creativity, and adaptability in uncertain environments [82].

As the process progresses, acquired knowledge is transformed to ensure its effective application [8,92]. This transformation allows stakeholders to respond adaptively to environmental changes [91,102], strengthening collective efforts to navigate uncertainties through collaboration [71]. The process aligns closely with the micro-foundations of dynamic capabilities [91], and the well-known elements of sensing, seizing, and reconfiguring. Stakeholders in this study demonstrated sensing by identifying shifts in customer demand and sustainability regulations, seizing opportunities by translating these insights into new hybrid configurations, and reconfiguring their approaches by integrating these adaptations into long-term routines and collaborations. However, unlike conventional dynamic capability processes confined within firm boundaries, these transformations in the case study occur across organizational boundaries through network-level collaboration, reinforcing recent evidence that coopetition fosters innovation and growth in technology-based firms [103].

In the final phase, transformed knowledge is integrated and applied in new contexts [87], resulting in new technological configuration and redefined sustainability objectives [6,48]. This phase reflects the iterative means and goal redefinition inherent in effectuation, while the sustainability orientation introduces a normative commitment to long-term ecological and social outcomes. This perspective challenges the original firm-centric assumption of effectuation theory [35], expanding its relevance to sustainability-driven, networked innovation in contexts of technological advancements.

These phases demonstrate that sustainable innovation is driven by iterative cycles of learning and collaboration rather than linear planning. Across all phases, the capacity of collaborating individuals to acquire, assimilate, transform, and exploit knowledge [29,36,87] moderates both phase transition and within-phase dynamics of sustainable innovation. Table 2 summarizes the key characteristics of each phase and their associated knowledge absorption mechanisms.

**Table 2:** Phases and characteristics of the sustainable innovation process.

Phases	Characteristics
**1 Awareness of obtainable resources and perception of uncertainty**	Recognition of external context and perception of uncertainty
	Acquisition of relevant knowledge to respond to customer needs
	Strategic adaptation to future trends
**2 Entrepreneurial goals for sustainable growth**	Open-mindedness to accept changes and new knowledge
	Assimilation of new knowledge to turn challenges into opportunities
	Advancing sustainability through technological innovation
**3 Stakeholder interaction to leverage opportunities**	Exchange knowledge with relevant stakeholders
	Transforming new knowledge to grasp identified opportunities
	Integrate knowledge to leverage opportunities for innovation
**4 Commitment to sustainable innovation**	Collaborative partnerships and continuous innovation to expand the market
	Formation of strategic partnerships and alliances
	Exploitation of technological knowledge to create value
**5 New means and new goals**	Achievement of new means and new goals
	Resources expand through sustainable innovation
	Converging on goals with a clear direction for innovation

### 5.2 Contributions

#### 5.2.1 Theoretical contributions.

This study makes several theoretical contributions to research on sustainable innovation, entrepreneurship, and knowledge-based perspectives. First, it advances effectuation theory by demonstrating how effectual principles operates within sustainability-oriented, multi-stakeholder innovation processes [104]. The findings confirm core effectual principles – particularly means-orientation, leveraging contingencies, and co-creation [34,35] – while extending and recontextualizing them beyond individual or firm-centric settings to networked contexts characterized by shared sustainability objectives. In doing so, the study contributes to a refined conceptualization of sustainability-oriented effectuation, in which adaptability and experimentation are directed toward ecological and social value creation [105–107].

Second, the study deepens understanding of absorptive capacity by conceptualizing knowledge absorption as a distributed and relational process rather than a purely organizational capability. By empirically linking specific dimensions of knowledge absorption to transitions between phases of sustainable innovation, the study elucidates how cognitive and relational mechanisms jointly enable sustainable innovation under uncertainty. This process-oriented perspective responds to calls for more dynamic explanations of how knowledge is mobilized and integrated within complex innovation ecosystems [108,21].

Third, the study refines the conceptual interplay between effectuation and absorptive capacity by demonstrating that awareness of obtainable resources and perception of uncertainty are not solely cognitive phenomena but are also shaped through boundary-spanning interactions. This finding aligns with emerging evidence that sustainable innovation in technology-intensive industries relies heavily on cognitive collaboration and cross-sectoral learning [103]. In this process, developers act as key boundary spanners, bridging institutional and technological domains by translating policy incentives and market signals into shared opportunity frames. These frames, in turn, establish the cognitive and relational foundations that enable subsequent goal formation and stakeholder engagement.

Finally, through the analytical generalization of a single, information-rich case, the study contributes to theory development by clarifying the mechanisms through which collaborative learning underpins sustainable innovation in technology-driven contexts [94,95]. The proposed framework extends existing literature on knowledge strategies and sustainable entrepreneurship [109,110]by demonstrating how boundary-spanning knowledge absorption supports coordinated experimentation, adaptive learning, and long-term commitment in sustainable innovation processes [111], particularly in technology-intensive contexts characterized by high levels of uncertainty [8,10,32].

#### 5.2.2 Practical implications.

From a practical perspective, this study offers actionable insights for entrepreneurs, managers, and policymakers engaged in technological innovation and sustainability. First, the proposed framework highlights the importance of deliberately cultivating knowledge absorption capabilities across organizational boundaries, emphasizing that sustainable innovation depends not only on technological expertise but also on the ability to integrate diverse knowledge sources [81,112]. Second, for practitioners, the results suggest that effectively, encouraging joint experimentation, supporting shared sensemaking processes, and balancing knowledge resource utilization can enhance both innovation performance and sustainability outcomes [8,83,113]. Additionally, the findings highlight the significance of the entrepreneurial network for acquiring and managing resources in sustainable innovation [114–116] and underscore the value of policy instruments that promote cross-sectoral collaboration and learning, thereby strengthening innovation ecosystems rather than individual firms.

#### 5.2.3 Limitations and further research.

Despite its contributions, this study has several limitations that suggest promising avenues for further research. First, the analysis does not explicitly examine the feedback loops associated with expanding resource cycles or converging goal constraints. Longitudinal research design could address this limitation by exploring how such feedback mechanisms operate overtime and how iterative learning processes reshape sustainable innovation trajectories. Second, the reliance on a single case limits statistical generalizability. Nevertheless, the study achieves analytical generalizability by developing theory grounded in rich and contextualized empirical evidence. Future research could strengthen and refine the proposed framework through comparative or multi-case studies conducted across diverse technological and institutional contexts. Third, future research could empirically test the propositions implied by the proposed framework, such as whether (a) Participants in sustainable innovation process operating in uncertain technology-driven contexts do not progress through phases sequentially but move back and forth; and (b) The four forms of knowledge absorption influence not only transition from one phase to another, but also the course of the phase itself. Multi-case qualitative research could provide deeper insights into how knowledge absorption facilitates sustainable innovation, while subsequent quantitative studies could statistically validate the proposed framework across diverse technological sectors or industries. Finally, future work could extend this line of inquiry by examining how digital transformation and emerging technologies – such as AI-driven design, advanced data analytics, and platform-based collaboration – reshape the mechanisms of knowledge absorption and stakeholder coordination identified in this study.

## 6 Conclusion

This study examined the sustainable innovation process in a technology-driven context through an integrated lens of effectuation and knowledge absorption. Drawing on an in-depth case analysis of the development of a hybrid marine engine, the study demonstrates how sustainability-oriented technological innovation unfolds through iterative experimentation, flexible decision-making, and continuous stakeholder engagement. In doing so, it identifies five distinct phases of the sustainable innovation process: (1) awareness of obtainable resources and perception of uncertainty, (2) entrepreneurial goals for sustainable growth, (3) stakeholder interaction to leverage opportunities, (4) commitment to sustainable innovation, and (5) new means and new goals. The findings further reveal that transitions between these phases are influenced by different dimensions of collaborating individuals’ knowledge absorption. Specifically, the transition from phase (1) to (2) is driven by knowledge acquisition; from (2) to (3) by knowledge assimilation; from (3) to (4) by knowledge transformation; and from (4) to (5) by knowledge exploitation. These insights underscore the importance of moving beyond deterministic and firm-centric approaches of technological innovation toward process-oriented frameworks that emphasize collaboration, experimentation, and shared learning. Overall, this study provides a theoretically grounded and empirically informed foundation for understanding how sustainable innovation emerges in complex, uncertain and technology-intensive environments.

## Supporting information

S1 File Appendix A: Representative interview quotes for five phases of the innovation process and four aspects of knowledge absorption. (DOCX)

## References

[1] Si S, Hall J, Suddaby R, Ahlstrom D, Wei J. Technology, entrepreneurship, innovation and social change in digital economics. Technovation. 2023;119:102484. doi: 10.1016/j.technovation.2022.102484

[2] Sharma R, Gupta H. Harmonizing sustainability in industry 5.0 era: Transformative strategies for cleaner production and sustainable competitive advantage. Journal of Cleaner Production. 2024;445:141118. doi: 10.1016/j.jclepro.2024.141118

[3] Ghobakhloo M, Iranmanesh M, Grybauskas A, Vilkas M, Petraitė M. Industry 4.0, innovation, and sustainable development: A systematic review and a roadmap to sustainable innovation. Bus Strat Env. 2021;30(8):4237–57. doi: 10.1002/bse.2867

[4] Kastelli I, Siokas G, Tsakanikas A. Entrepreneurial Absorptive Capacity As Enabler of Knowledge Intensive Entrepreneurship: An Empirical Investigation. J Knowl Econ. 2023;15(2):9667–98. doi: 10.1007/s13132-023-01465-9

[5] Bailetti T. Technology entrepreneurship: overview, definition, and distinctive aspects. Technology Innovation Management Review. 2012;5:12.

[6] Chung D, Jung H, Lee Y. Investigating the relationship of high-tech entrepreneurship and innovation efficacy: The moderating role of absorptive capacity. Technovation. 2022;111:102393. doi: 10.1016/j.technovation.2021.102393

[7] Garud R, Karnøe P. Bricolage versus breakthrough: distributed and embedded agency in technology entrepreneurship. Research Policy. 2003;32(2):277–300. doi: 10.1016/s0048-7333(02)00100-2

[8] Audretsch DB, Fiedler A. Bringing the knowledge spillover theory of entrepreneurship to circular economies: Knowledge and values in entrepreneurial ecosystems. International Small Business Journal: Researching Entrepreneurship. 2023;42(4):480–505. doi: 10.1177/02662426231218357

[9] Mosey S, Guerrero M, Greenman A. Technology entrepreneurship research opportunities: insights from across Europe. J Technol Transf. 2016;42(1):1–9. doi: 10.1007/s10961-015-9462-3

[10] Urbano D, Guerrero M, Ferreira JJ, Fernandes CI. New technology entrepreneurship initiatives: Which strategic orientations and environmental conditions matter in the new socio-economic landscape?. J Technol Transf. 2018;44(5):1577–602. doi: 10.1007/s10961-018-9675-3

[11] Kulkov I, Ivanova-Gongne M, Bertello A, Makkonen H, Kulkova J, Rohrbeck R, et al. Technology entrepreneurship in healthcare: Challenges and opportunities for value creation. Journal of Innovation & Knowledge. 2023;8(2):100365. doi: 10.1016/j.jik.2023.100365

[12] Zhang G, Peng X, Li J. Technological entrepreneurship and policy environment: a case of China. Journal of Small Business and Enterprise Development. 2008;15(4):733–51. doi: 10.1108/14626000810917834

[13] Myers A, Albats E. The Challenges and Strategic Solutions of Emerging Technology Entrepreneurship: A Systematic Literature Review. In: Proceedings of the Annual Hawaii International Conference on System Sciences, 2024. doi: 10.24251/hicss.2024.653

[14] Liu J, Hu J, Wu D, Chen J. Patterns of technological entrepreneurship and their determinants: Evidence from technology-based manufacturing firms in China. Entrepreneurship Research Journal. 2024;14:1259–78.

[15] Wang X, Wang Z, Zhang M. Knowledge Workers, Innovation Linkages and Knowledge Absorption: An Interactive Mechanism Study. J Knowl Econ. 2024;15(4):19460–89. doi: 10.1007/s13132-023-01709-8

[16] He F, Miao X, Wong CWY, Lee S. Contemporary corporate eco-innovation research: A systematic review. Journal of Cleaner Production. 2018;174:502–26. doi: 10.1016/j.jclepro.2017.10.314

[17] Carrión-Flores CE, Innes R. Environmental innovation and environmental performance. Journal of Environmental Economics and Management. 2010;59(1):27–42. doi: 10.1016/j.jeem.2009.05.003

[18] Varadarajan R. Innovating for sustainability: a framework for sustainable innovations and a model of sustainable innovations orientation. J of the Acad Mark Sci. 2015;45(1):14–36. doi: 10.1007/s11747-015-0461-6

[19] Mutambik I. Digital Transformation as a Driver of Sustainability Performance—A Study from Freight and Logistics Industry. Sustainability. 2024;16(10):4310. doi: 10.3390/su16104310

[20] Sossa JW, Lopez Montoya OH, Acosta Prado JC. Determinants of a sustainable innovation system. Business Strategy and the Environment. 2021;30(2):1345–56.

[21] Afeltra G, Alerasoul SA, Strozzi F. The evolution of sustainable innovation: from the past to the future. EJIM. 2021;26(2):386–421. doi: 10.1108/ejim-02-2021-0113

[22] Boons F, Lüdeke-Freund F. Business models for sustainable innovation: state-of-the-art and steps towards a research agenda. Journal of Cleaner Production. 2013;45:9–19. doi: 10.1016/j.jclepro.2012.07.007

[23] Klewitz J, Hansen EG. Sustainability-oriented innovation of SMEs: a systematic review. Journal of Cleaner Production. 2014;65:57–75. doi: 10.1016/j.jclepro.2013.07.017

[24] Adams R, Jeanrenaud S, Bessant J, Denyer D, Overy P. Sustainability‐oriented Innovation: A Systematic Review. Int J Management Reviews. 2015;18(2):180–205. doi: 10.1111/ijmr.12068

[25] Schaltegger S, Hansen EG, Lüdeke-Freund F. Business models for sustainability: origins, present research, and future avenues. Organization & Environment. 2016;29(1):3–10.

[26] Román S, Bodenstab S, Sánchez-Siles LM. Corporate tensions and drivers of sustainable innovation: a qualitative study in the food industry. EJIM. 2021;25(4):925–47. doi: 10.1108/ejim-11-2020-0469

[27] Longoni A, Cagliano R. Sustainable Innovativeness and the Triple Bottom Line: The Role of Organizational Time Perspective. J Bus Ethics. 2016;151(4):1097–120. doi: 10.1007/s10551-016-3239-y

[28] Adomako S, Tran MD. Scaling up sustainable innovation: Stakeholder ties, eco‐product innovation, and new product performance. Sustainable Development. 2023;32(1):624–34. doi: 10.1002/sd.2700

[29] Zahra SA, George G. Absorptive capacity: a review, reconceptualization, and extension. The Academy of Management Review. 2002;27(2):185. doi: 10.2307/4134351

[30] Gruber M, MacMillan IC, Thompson JD. Look Before You Leap: Market Opportunity Identification in Emerging Technology Firms. Management Science. 2008;54(9):1652–65. doi: 10.1287/mnsc.1080.0877

[31] Shane S. Technological Opportunities and New Firm Creation. Management Science. 2001;47(2):205–20. doi: 10.1287/mnsc.47.2.205.9837

[32] Autio E, Kenney M, Mustar P, Siegel D, Wright M. Entrepreneurial innovation: The importance of context. Research Policy. 2014;43(7):1097–108.

[33] Ferreira JJM, Ferreira FAF, Fernandes CIMAS, Jalali MS, Raposo ML, Marques CS. What do we [not] know about technology entrepreneurship research? International Entrepreneurship and Management Journal. 2016;12:713–33.

[34] Sarasvathy SD, Dew N. New market creation through transformation. J Evol Econ. 2005;15(5):533–65. doi: 10.1007/s00191-005-0264-x

[35] Sarasvathy SD. Causation and Effectuation: Toward a Theoretical Shift from Economic Inevitability to Entrepreneurial Contingency. The Academy of Management Review. 2001;26(2):243. doi: 10.2307/259121

[36] Lowik S, Kraaijenbrink J, Groen AJ. Antecedents and effects of individual absorptive capacity: a micro-foundational perspective on open innovation. JKM. 2017;21(6):1319–41. doi: 10.1108/jkm-09-2016-0410

[37] Cohen WM, Levinthal DA. Absorptive Capacity: A New Perspective on Learning and Innovation. Administrative Science Quarterly. 1990;35(1):128. doi: 10.2307/2393553

[38] Parolin G, McAloone TC, Pigosso DCA. How can technology assessment tools support sustainable innovation? A systematic literature review and synthesis. Technovation. 2024;129:102881. doi: 10.1016/j.technovation.2023.102881

[39] Scuotto V, Alfiero S, Cuomo MT, Monge F. Knowledge management and technological innovation in family SMEs context. JKM. 2023;28(3):789–98. doi: 10.1108/jkm-04-2023-0281

[40] Callerstig A-C, Lindvert M, Ljunggren EC, Breivik-Meyer M, Alsos GA, Balkmar D. Contextualising gender policy in tech entrepreneurship: a cross national and multiple-level analysis. IJEBR. 2024;30(7):1678–97. doi: 10.1108/ijebr-04-2023-0422

[41] McMullen JS, Dimov D. Time and the Entrepreneurial Journey: The Problems and Promise of Studying Entrepreneurship as a Process. J Management Studies. 2013;50(8):1481–512. doi: 10.1111/joms.12049

[42] Cillo V, Petruzzelli AM, Ardito L, Del Giudice M. Understanding sustainable innovation: A systematic literature review. Corp Soc Responsibility Env. 2019;26(5):1012–25. doi: 10.1002/csr.1783

[43] Khurana I, Dutta DK, Schenkel MT. Crisis and arbitrage opportunities: The role of causation, effectuation and entrepreneurial learning. International Small Business Journal: Researching Entrepreneurship. 2021;40(2):236–72. doi: 10.1177/02662426211061679

[44] Perry JT, Chandler GN, Markova G. Entrepreneurial Effectuation: A Review and Suggestions for Future Research. Entrepreneurship Theory and Practice. 2012;36(4):837–61. doi: 10.1111/j.1540-6520.2010.00435.x

[45] Chandler GN, DeTienne DR, McKelvie A, Mumford TV. Causation and effectuation processes: A validation study. Journal of Business Venturing. 2011;26(3):375–90. doi: 10.1016/j.jbusvent.2009.10.006

[46] Yoshino M, Sadlek B, Yarime M, Ali A. Knowledge absorption pathways for eco-innovation: an empirical analysis of small and medium-sized enterprises in the European Union. EJIM. 2023;28(2):426–53. doi: 10.1108/ejim-02-2023-0136

[47] Brieger SA, De Clercq D. Entrepreneurs’ individual-level resources and social value creation goals: The moderating role of cultural context. International Journal of Entrepreneurial Behaviour and Research. 2019;25:193–216.

[48] García-Hurtado D, Devece C, Zegarra-Saldaña PE, Crisanto-Pantoja M. Ambidexterity in entrepreneurial universities and performance measurement systems. A literature review. International Entrepreneurship and Management Journal. 2024; 20:345–66.

[49] Wernerfelt B. A resource‐based view of the firm. Strategic Management Journal. 1984;5(2):171–80. doi: 10.1002/smj.4250050207

[50] Lockett A, Thompson S. The resource-based view and economics. Journal of Management. 2001;27(6):723–54. doi: 10.1177/014920630102700608

[51] Brown C, Hooley T, Wond T. Building career capital: developing business leaders’ career mobility. CDI. 2020;25(5):445–59. doi: 10.1108/cdi-07-2019-0186

[52] Gabrielsson J, Politis D. Career motives and entrepreneurial decision-making: examining preferences for causal and effectual logics in the early stage of new ventures. Small Bus Econ. 2009;36(3):281–98. doi: 10.1007/s11187-009-9217-3

[53] Han W, Li X, Zhu W, Lu R, Zu X. Knowledge digitization and high-tech firm performance: A moderated mediation model incorporating business model innovation and entrepreneurial orientation. Technology in Society. 2024;77:102536. doi: 10.1016/j.techsoc.2024.102536

[54] Wang Y, Zhou Y. Innovation network, knowledge absorption ability, and technology innovation performance--An empirical analysis of China’s intelligent manufacturing industry. PLoS One. 2023;18(11):e0293429. doi: 10.1371/journal.pone.0293429 37948423 PMC10637672

[55] Nigra M, Bossink B. Cooperative Learning in Green Building Demonstration Projects: Insights from 30 Innovative and Environmentally Sustainable Demonstrations around the World. J Constr Eng Manage. 2023;149(4). doi: 10.1061/jcemd4.coeng-12881

[56] Blanchett M, Rubach MJ, Duggins R. Social capital and success of minority entrepreneurs. Journal of Business Diversity. 2019;19:108–22.

[57] Van den Bosch FAJ, Volberda HW, de Boer M. Coevolution of Firm Absorptive Capacity and Knowledge Environment: Organizational Forms and Combinative Capabilities. Organization Science. 1999;10(5):551–68. doi: 10.1287/orsc.10.5.551

[58] Tryba A, Fletcher D. How shared pre-start-up moments of transition and cognitions contextualize effectual and causal decisions in entrepreneurial teams. Small Bus Econ. 2019;54(3):665–88. doi: 10.1007/s11187-019-00148-7

[59] Todorova G, Durisin B. Absorptive capacity: valuing a reconceptualization. Academy of Management Review. 2007.

[60] Hessels J, van Gelderen M, Thurik R. Entrepreneurial aspirations, motivations, and their drivers. Small Bus Econ. 2008;31(3):323–39. doi: 10.1007/s11187-008-9134-x

[61] Nathani N, Dwivedi G. Influence of Technology Entrepreneurship on Entrepreneurial Intentions: A Cross Country Analysis. SSRN Journal. 2019. doi: 10.2139/ssrn.3319889

[62] Dutta DK, Gwebu KL, Wang J. Personal innovativeness in technology, related knowledge and experience, and entrepreneurial intentions in emerging technology industries: a process of causation or effectuation?. International Entrepreneurship and Management Journal. 2015;11:529–55.

[63] Gustafsson V. Entrepreneurial decision-making: Individuals, tasks and cognitions. Edward Elgar Publishing; 2006.

[64] Chen A, Lin Y, Mariani M, Shou Y, Zhang Y. Entrepreneurial growth in digital business ecosystems: an integrated framework blending the knowledge-based view of the firm and business ecosystems. J Technol Transf. 2023;48(5):1628–53. doi: 10.1007/s10961-023-10027-9

[65] Chesbrough HW. The era of open innovation. MIT Sloan Management Review. 2003;44:35–42.

[66] Ge S, Liu X. The role of knowledge creation, absorption and acquisition in determining national competitive advantage. Technovation. 2022;112:102396. doi: 10.1016/j.technovation.2021.102396

[67] Ketchen DJ Jr, Ireland RD, Snow CC. Strategic entrepreneurship, collaborative innovation, and wealth creation. Strategic Entrepreneurship. 2007;1(3–4):371–85. doi: 10.1002/sej.20

[68] Greer CR, Lei D. Collaborative Innovation with Customers: A Review of the Literature and Suggestions for Future Research*. Int J Management Reviews. 2011;14(1):63–84. doi: 10.1111/j.1468-2370.2011.00310.x

[69] Závodská A, Šramová V. Collaboration and Knowledge Sharing as a Key to Success of Entrepreneurial Ecosystem. Communications in Computer and Information Science. Springer International Publishing; 2018. p. 128–39. doi: 10.1007/978-3-319-95204-8_12

[70] Wiltbank R, Dew N, Read S, Sarasvathy SD. What to do next? The case for non‐predictive strategy. Strategic Management Journal. 2006;27(10):981–98. doi: 10.1002/smj.555

[71] Fauzi TH, Harits B, Danial DM, Komariah K. Adaptive Strategies of External Environmental Effects in Digital Entrepreneurship in the Strategic Management Perspective. AJIS. 2020;9(3):38. doi: 10.36941/ajis-2020-0040

[72] Wiltbank R, Read S, Dew N, Sarasvathy SD. Prediction and control under uncertainty: Outcomes in angel investing. Journal of Business Venturing. 2009;24(2):116–33. doi: 10.1016/j.jbusvent.2007.11.004

[73] Bommert B. Collaborative innovation in the public sector. International Public Management Review. 2010;11:15–33.

[74] Chesbrough HW, Appleyard MM. Open Innovation and Strategy. California Management Review. 2007;50(1):57–76. doi: 10.2307/41166416

[75] Bacchiocchi A, Bellocchi A, Giombini G. Green investment challenges in European firms: Internal vs. external resources. Sustainability. 2024;16(2):496.

[76] Chung D, Jung H, Lee Y. Investigating the relationship of high-tech entrepreneurship and innovation efficacy: The moderating role of absorptive capacity. Technovation. 2022;111:102393. doi: 10.1016/j.technovation.2021.102393

[77] Van Mumford J, Zettinig P. Co-creation in effectuation processes: A stakeholder perspective on commitment reasoning. Journal of Business Venturing. Journal of Business Venturing. 2022;37(4):106209. doi: 10.1016/j.jbusvent.2022.106209

[78] Zhang SX, Van Burg E. Advancing entrepreneurship as a design science: developing additional design principles for effectuation. Small Bus Econ. 2019;55(3):607–26. doi: 10.1007/s11187-019-00217-x

[79] Hayton JC, Zahra SA. Venture team human capital and absorptive capacity in high technology new ventures. IJTM. 2005;31(3/4):256. doi: 10.1504/ijtm.2005.006634

[80] Kirschning R, Mrożewski M. The role of entrepreneurial absorptive capacity for knowledge spillover entrepreneurship. Small Bus Econ. 2022;60(1):105–20. doi: 10.1007/s11187-022-00639-0

[81] Lichtenthatler U. Absorptive capacity, environmental turbulence, and the complementarity of organizational learning processes. The Academy of Management Journal. 2009;52:822–46.

[82] Liu X, Yuan Y, Sun R, Zhao C, Zhao D. Influence of entrepreneurial team knowledge conflict on ambidextrous entrepreneurial learning— a dual-path perspective of entrepreneurial resilience and fear of failure. Journal of Innovation & Knowledge. 2023;8(3):100389. doi: 10.1016/j.jik.2023.100389

[83] Yin J, Li Y, Ma Z, Chen Z, Guo G. Impact of entrepreneurship on technological innovation in the digital age: a knowledge management perspective. JKM. 2024;28(9):2750–72. doi: 10.1108/jkm-07-2023-0602

[84] Prasetyoa PE, Siswantarib H. Technology Absorption in Entrepreneurial Aspirations and Capabilities. Technology. 2020;14(3).

[85] Tukamuhabwa B, Namagembe S. Participation of women-owned SMEs in public procurement: the role of entrepreneurial orientation and knowledge management orientation. JOPP. 2023;23(3/4):273–96. doi: 10.1108/jopp-01-2023-0002

[86] Hung S-C, Chu Y-Y. Stimulating new industries from emerging technologies: challenges for the public sector. Technovation. 2006;26(1):104–10. doi: 10.1016/j.technovation.2004.07.018

[87] Wang M, Wang Y, Mardani A. Empirical analysis of the influencing factors of knowledge sharing in industrial technology innovation strategic alliances. Journal of Business Research. 2023;157:113635. doi: 10.1016/j.jbusres.2022.113635

[88] Rae D, Carswell M. Using a life‐story approach in researching entrepreneurial learning: the development of a conceptual model and its implications in the design of learning experiences. Education + Training. 2000;42(4/5):220–8. doi: 10.1108/00400910010373660

[89] Ul-Durar S, Awan U, Varma A, Memon S, Mention A-L. Integrating knowledge management and orientation dynamics for organization transition from eco-innovation to circular economy. JKM. 2023;27(8):2217–48. doi: 10.1108/jkm-05-2022-0424

[90] Gebauer H, Worch H, Truffer B. Absorptive capacity, learning processes and combinative capabilities as determinants of strategic innovation. European Management Journal. 2012;30(1):57–73. doi: 10.1016/j.emj.2011.10.004

[91] Teece DJ. Explicating dynamic capabilities: the nature and microfoundations of (sustainable) enterprise performance. Strategic Management Journal. 2007;28(13):1319–50. doi: 10.1002/smj.640

[92] Lane PJ, Koka BR, Pathak S. The Reification of Absorptive Capacity: A Critical Review and Rejuvenation of the Construct. AMR. 2006;31(4):833–63. doi: 10.5465/amr.2006.22527456

[93] Eisenhardt KM. Building theories from case study research. Acad Manag Rev. 1989;14:532–50.

[94] Eisenhardt KM, Graebner ME. Theory Building From Cases: Opportunities And Challenges. AMJ. 2007;50(1):25–32. doi: 10.5465/amj.2007.24160888

[95] Yin RK. Case study research: design and methods. 5th ed. SAGE Publication; 2014.

[96] Dubois A, Gadde L-E. Systematic combining: an abductive approach to case research. Journal of Business Research. 2002;55(7):553–60. doi: 10.1016/s0148-2963(00)00195-8

[97] Gioia DA, Corley KG, Hamilton AL. Seeking qualitative rigor in inductive research: notes on the Gioia methodology. Organiz Res Methods. 2013;16(1):15–31.

[98] Hancock DR, Algozzine B. Doing case study research: A practical guide for beginning researchers. 6 ed. New York and London: Teachers College Press; 2006.

[99] Fisher G. Effectuation, Causation, and Bricolage: A Behavioral Comparison of Emerging Theories in Entrepreneurship Research. Entrepreneurship Theory and Practice. 2012;36(5):1019–51. doi: 10.1111/j.1540-6520.2012.00537.x

[100] Jiang Y, Rüling C-C. Opening the Black Box of Effectuation Processes: Characteristics and Dominant Types. Entrepreneurship Theory and Practice. 2017;43(1):171–202. doi: 10.1177/1042258717744204

[101] Fan J, Su J, Sindakis S. Customer Need Knowledge Facilitates Market Opportunity Recognition Through Absorptive Capacity and Technological Knowledge: Evidence from the IT Sector in China. J Knowl Econ. 2023;15(1):2192–217. doi: 10.1007/s13132-023-01210-2

[102] Asad M, Majali T, Aledeinat M, Abdelkarim Almajali D, Akhorshaideh AHO. Green entrepreneurial orientation for enhancing SMEs financial and environmental performance: Synergetic moderation of green technology dynamism and knowledge transfer and integration. Cogent Business & Management. 2023;10(3). doi: 10.1080/23311975.2023.2278842

[103] Salamzadeh A, Dana L-P, Rastgoo N, Hadizadeh M, Mortazavi SM. The Role of Coopetition in Fostering Innovation and Growth in New Technology-based Firms: A Game Theory Approach. BAR, Braz Adm Rev. 2024;21(1). doi: 10.1590/1807-7692bar2024230097

[104] Brun A, Ciccullo F. Factors affecting sustainability‐oriented innovation in the leather supply chain. Strategic Change. 2022;31(3):305–21. doi: 10.1002/jsc.2500

[105] Salamzadeh A, Hadizadeh M, Rastgoo N, Rahman MdM, Radfard S. Sustainability-Oriented Innovation Foresight in International New Technology Based Firms. Sustainability. 2022;14(20):13501. doi: 10.3390/su142013501

[106] Hajdukiewicz A, Pera B. Eco-innovation in the European Union: Challenges for catching-up economies. Entrepreneurial Business and Economics Review. 2023;11(1):145–64.

[107] Zighan S, Abuhussein T, Al-Zu’bi ZB, Dwaikat NY. A qualitative exploration of factors driving sustainable innovation in small-and medium-sized enterprises in Jordan. Journal of Enterprising Communities: People and Places in the Global Economy. 2024;18(2):372–91.

[108] Busenitz LW, Plummer LA, Klotz AC, Shahzad A, Rhoads K. Entrepreneurship Research (1985-2009) and the Emergence of Opportunities. Entrepreneurship: Theory and Practice. 2014;38:981–1000.

[109] Ferreira JJ, Cruz B, Veiga PM, Ribeiro-Soriano D. Knowledge strategies and digital technologies maturity: effects on small business performance. Entrepreneurship & Regional Development. 2022;36(1–2):36–54. doi: 10.1080/08985626.2022.2159544

[110] Cuvero M, Granados ML, Pilkington A, Evans R. Start‐ups’ use of knowledge spillovers for product innovation: the influence of entrepreneurial ecosystems and virtual platforms. R & D Management. 2022;53(4):584–602. doi: 10.1111/radm.12567

[111] Makhloufi L. Do knowledge sharing and big data analytics capabilities matter for green absorptive capacity and green entrepreneurship orientation? Implications for green innovation. Industrial Management and Data Systems. 2024; 124:978–1004.

[112] Cristo-Andrade S, Ferreira JJ, Teixeira A, McDowell WC. Knowledge spillovers in business intelligence organisations: a strategic entrepreneurship perspective. Int Entrep Manag J. 2023;20(2):733–59. doi: 10.1007/s11365-023-00896-9

[113] Stets JE, Cast AD. Resources and Identity Verification from an Identity Theory Perspective. Sociological Perspectives. 2007;50(4):517–43. doi: 10.1525/sop.2007.50.4.517

[114] Ramachandran K, Ray S. Networking and new venture resource strategies. The Journal of Entrepreneurship. 2006;15:145–68.

[115] Zane LJ, DeCarolis DM. Social networks and the acquisition of resources by technology-based new ventures. Journal of Small Business & Entrepreneurship. 2016;28(3):203–21. doi: 10.1080/08276331.2016.1162048

[116] Shane S. Encouraging university entrepreneurship? The effect of the Bayh-Dole Act on university patenting in the United States. Journal of Business Venturing. 2004;19(1):127–51.

