# Supplementary material for: Enhancing sustainable innovation through collaborative knowledge absorption: Insights from the development of a hybrid marine engine
Source: PLoS One. 2026 Apr 15;21(4):e0346929. doi: 10.1371/journal.pone.0346929 (PMC13082694; doi:10.1371/journal.pone.0346929)
Supplement: S1 File — (DOCX) [file pone.0346929.s001.docx]

**Appendix A: Representative interview quotes for five phases of the innovation process and four aspects of knowledge absorption**

| **Phase 1 Awareness of obtainable resources and perception of uncertainty** | | |
| --- | --- | --- |
| **Characteristics** | **Key informant terms** | **Representative quotes from interviewees** |
| Perception of challenges in contexts | Environmental and infrastructural challenges towards green energy | (S3) "Here in marina, all infrastructure is old. If we put ten electric boats here, probably the whole marina will collapse." (S4) "The emission regulations are developing according to the world situation. However, on the sea there are still many vessels which are driven by engines which are from an older date, much older than the ones which are supported with the emission regulations nowadays." (S9) "To conclude, the general issue here is a very old infrastructure." (S10) "Hydrogen is one of the options for storing energy." (S12) "The current plan for revitalization is active, although it is not yet public. The main measures for sea transport are the port reconstruction and transition for fuel." |
|  | Recognize value of potential technological opportunities | (S6) "Since it's a new technique, we started talking about electric propulsion for boats. The first step towards accepting hybrid propulsion in a boat is today" (S6) "The advancement of technologies today is not solely functionality, but functionality with operability. New technologies are complex, but they are safe to work with." (S6) "Iridium might work at 150 degrees as well, where lower temperatures imply higher efficiency. With that technology hydrogen can be stored and extracted just like fuel, stored in canisters." (S10) "hydrogen combined in a storage with CO2, called synthetic methanol, we see as an option. Ammonia we see as an option as well." (S13) "For solid state, scaling up is a challenge. I noticed that companies like BMW and Mercedes are betting on this type of technology." |
|  | Market scanning: meeting diverse customer demands | (S2) "There are different demand inquiries regarding propulsion systems, where hybrid systems are flexible in answering to those demand inquiries." S3) "The system should be balanced, taking into account the customer’s needs." (S3) "People are looking into 48 feet vessels and they want them to be hybrid too, but only if the boat will go 20-25 knots in diesel mode."  (S4) "The company “company name” is more than two years in the assembling of hybrid solutions. The main reason was the market demand, the demand of one of our most important client and market trends." (S6) "When you have two systems in a hybrid engine, sometimes customers would want to use each system for different things and separate purposes. The appetite of the market increases. The customer may always propose different solutions which he sees as improvements, motivating you to act upon them and this is exactly the development we are looking for." (S9) "It is a too expensive investment if the marina is going to charge only two vessels. If demand changes, of course our viewpoint will change too." (S10) "The wealthy clients we have are actually pushing the boundaries and research and development for these vessels. They are running ahead of the commercial part of business." |
| Awareness of obtainable resources | Clear self-identification: innovation to provide comfort | (S3) "We are not developing the battery itself. " (S5) "What I can offer as an entrepreneur to my worker is, at our company he can learn a lot if he wants, he can earn money if he wants. " (S4) "I am aware that our engines not only comply, but in certain cases surpass the emission regulations on engines. Our engines comply with IMO Tier 2, EPA Tier 3 and EU RCD 2 emission regulations." (S6) "There are always some limiting factors, what we can do and what we can’t." (S7) "Our basic product is the engine and on that ground we provide a system, the hybrid engine." (S10) "Additional energy provider just gives the freedom the client is looking for." |
|  | Relevant knowledge: from environmental considerations to technological fusion | (S10) "We know how to optimize the hull shape and the appendices of the rudder and the design of sailing solutions for wind assisted vessels, so sailing yachts." (S11) "Hybrid is a very wide understanding. We can claim we have the electrical motor driven by the electrical generator, on the other hand we have the main engine, which is connected straight to the propulsion, it’s a hybrid." (S11) "Hybrid means you can drive the ship through an electrical motor, or straight from the diesel engine." |
|  | Leverage network resources to facilitate innovation | (S1) "When you have a project, there is a higher chance of retrieving funding. Especially for electric and hybrid, since both are more environmentally conscious variants and better than a diesel engine." (S3) "The government is ready to build a special charging point, it’s a special approach. For them it’s business." (S5) "Every stakeholder is focused on its industry and its products. If the client’s demand and market knowledge, our knowledge and the electric engine supplier’s knowledge unite, we can achieve a lot." (S8) "When we got sufficient funding, we immediately progressed in that direction of electrification." (S8) "The funding for the electrical connection on the dock was founded by our ministry of environmental protection, while for the rest we applied to EU funds." |
| **Phase 2 Entrepreneurial goals for sustainable growth** | | |
| **Characteristics** | **Key informant terms** | **Representative quotes from interviewees** |
| Proactive aspiration and flexibility | Recognition of advantages and disadvantages | (S1) "My opinion is that currently electric energy is not as green as it is considered, because it is mostly generated from non-renewable energy sources. The counterpart would be generating electricity from a clean source for vessel propulsion." (S2) "Their first advantage is their adaptability to different loads. Alongside, their flexibility to different vessels, propulsion types and loads." (S2) "The advantage is certainly a smaller rate of fuel consumption, because the engine’s flexibility allows it to work more optimally in a specific working point. Consequently, the emission of toxic gases is decreased. If the engine works within a better regime, that will result in an improved engine lifetime and longer maintenance periods." (S3) "Electric propulsion at sea means you have limited your range. This is the only main disadvantage of an electric engine." (S3) "Hybrid system advantage compared to electric is the non-limited range." (S4) "The main advantage of electric engine is its cleanliness and its emission" (S10) "The diesel has a far greater density than anything we can come up with, whether it's hydrogen, ammonia or synthetic methanol. The energy density of diesel is not achievable yet." |
|  | Aspirations: entrepreneurial vision and technical innovation | (S5) "When I was young and started working, my motivation was achieving something. " (S5) "The goal of the company is to have unity and a common goal, the company and team benefit." (S6) "As we were born within this surrounding, this technology oriented us towards boat building." (S6) "We are developing these new systems, which are systems with a permanent magnet." (S7) "One young engineer got his desk in an office, but after three days he moved himself to production. He is then in constant communication with the other employees and can monitor everything efficiently. There is feedback. We live with the product, it is not far from us." (S10) "You have to design a really good sailing vessel, and you need to use your hydrogenation more often." |
|  | Response to market trends: flexibility and adaptability | (S3) "They build now, the market grows and people will charge." (S3) "In 2010 was the economic crisis and people were looking for cheaper solutions with less consumption, but everyone wants to be green and no one wants to pay for it." (S11) "Most of the needs for things like this is raised by awareness. More and more people are demanding." |
|  | Open-minded: collaborative initiative and proactivity | (S5) "Okay, if someone does their work very well, I respect it. But normally, I am always looking for someone above the average who wants to achieve more." (S7) "We have a competitive advantage that we have been on the market for a very long time. We have a lot of experience before other larger systems, because for larger systems this isn’t as profitable. It is too expensive and complex however, for industrialization." (S8) "There were sufficient funds, but it’s always the question how interested the stakeholders were." |
| Advancing sustainability through technological innovation | Pursuing sustainable and renewable energy solutions | (S6) "Today is the trend of circular technologies." (S6) "One part is regarding the work of the system and the other of the decomposing of the system. More or less, in our engines and diesel engines, not batteries, everything is recyclable." (S8) "We don’t pollute anymore, since we are in a specific zoological natural park. The ecosystem in which we are is one step above national parks and we are really paying attention to that." (S11) "The goal is to be carbon neutral by 2050. It is quite ambitious, even the goal for 2030 to cut the emission by half. This is again differently applied to different types of vessels. For the bigger vessels, the effect will be the biggest." (S11) "Not all electric things are sustainable. How do you recharge? If your power source is from a coal powered plant, then it really doesn’t make sense. It needs to come from renewable." (S13) "There are six projects where we speak about sustainability and alternatives against fossil fuels. We haven’t decided what is the best and what will be applicable for which application, but at least we have six projects in development." |
|  | Continuous improvement and technological evolution | (S5) "There is always a place for improvement of the team, tools, technology and processes." (S6) "The source of energy and batteries will evolve a lot in the future. Supercapacitors are a new avenue." (S6) "Nothing is perfect on this world, because if we would have developed a perfect engine the overall technology development would stop." (S6) "This technique has space for improvement, because it is not efficient yet." (S6) "What we do in parallel hybrids today has its flaws, but in comparison to our concurrent it is perfect and the market accepts it. We just need to develop it." |
| **Phase 3 Stakeholder interaction to leverage opportunities** | | |
| **Characteristics** | **Key informant terms** | **Representative quotes from interviewees** |
| Foster internal collaboration and empower teams | Integration of technical expertise and employee engagement | (S7) "If I need to bring something to the employees, we receive it back." (S7) "The product you get is a result of technical work, development of the company and similar. Behind this project is a technologist." (S8) "Employees which are interested in and are working on getting funding for the right causes need to have sufficient manpower." |
|  | Effective organizational structure and constructive feedback | (S5) "There is feedback in the company. One always insists on feedback because without feedback there is no analysis of business elements. If there is no quality feedback, there is no quality analysis of work." (S5) "The leadership style in the company is vertical because it is a micro business. There is no other way to structure the business. In a small company, the most common way is the vertical leadership where a few specific positions are profiled out." (S5) "I think that the company is very well organised and its work being done sufficiently." (S7) "The structure of the company and monitoring of sales imposes subordinates and superiors. There needs to be structure to work efficiently with larger companies. Despite the small size, for every task we have a man and a half working on it." |
| Building partnerships through external communication | Strategic resource management and stakeholder engagement | (S8) "Stakeholders need to have sufficient manpower. " |
|  | Customer education and effective communication | (S3) "Many customers don’t have a clue what a hybrid or electric engine is, how does it work and what is the advantage and what is the disadvantage." (S3) "An old man ordered a year ago an electric boat, because he was tired of using diesel or gasoline for his entire life. In the beginning he complained about the charging, but then he realized he can charge overnight." (S4) "We have feedback about customer satisfaction regarding the use of the electric engine as propulsion. The client is satisfied." (S6) "After you ship an engine, each customer sees something which might be added upon." (S7) "Also, there might be the owner or captain which has specific requirements. Therefore, you need to follow upon these requirements as an engineer." |
| **Phase 4 Commitment to sustainable innovation** | | |
| **Characteristics** | **Key informant terms** | **Representative quotes from interviewees** |
| Strategic innovation to expand market | Explore diverse energy solutions to reach the goals | (S2) "Currently there are twenty to fifty ways to store hydrogen, but none of them has been renowned as universal and efficient. There is a proposal for storing hydrogen as an emulsion." (S13) "It’s all related we need more power, through solid state and hydrogen. " (S13) "I think in the future you will have different solutions for different applications, and it will be a race against conventional lithium battery i.e. solid state and hydrogen. Hydrogen is expensive, inefficient, has some disadvantages in structure. Many things limit the implementation of hydrogen fuel cells. Second option is solid state. The third option is lithium. " |
|  | Set new goals to meet market demands | (S3) "However, for this we need to design the new electric unit which can hold at least 400 HP which could be transferred to the electric motor and propulsion system. It should be at least holding this power, mechanically wise."  (S7) "Currently we are producing a variable speed generator, a completely new aspect. The demand for 500-600 kW is growing." (S11) "The new ship will also have batteries. We can regenerate the power when we sail on the sails. Energy can be produced and stored to batteries and when needed, used for propulsion. This is the revolution part." (S11) "We optimize by using the main engines. To keep the generator under the optimal load range. Our target was to reduce the speed of the ship." |
|  | Scaling the business: meet diverse market needs | (S3) "We applied this solution to the pleasure market. We can now drive electric in any moment, or we can use the energy we stored, which was generated during the diesel propulsion navigation. This is one of the main concepts of the hybrid system. " (S3) "We try to make our system balanced so it can charge overnight."(S4) "The first step would be, to at least, do a more contemporary change of propulsions inside the boats. There are many opportunities to take care of the environment." (S7) "You use this innovation for one purpose, but then the market broadens... By broadening the market, you meet different specifics the market requires. " |
| Collaborative partnerships for market growth | Refine technology: advancements in power generation and storage | (S3) "We can install a range extender, another diesel generator which means less noise, less consumption, but with all these features you need to have a fuel tank and maintenance on the diesel engine. " (S6) "There is a variant of portable energy. A liter of fuel has a lot of energy. A kilogram of hydrogen has three times the energy of a kilogram of fuel. The kilogram of batteries has maybe ten times less than the kilogram of fuel." |
|  | Strengthen market expansion efforts through collaborative partnerships | (S5) "This assembly is achieved by multiple stakeholders contributing to the assembly. For assembling a hybrid, we cannot simply ship a diesel engine. We need to collaborate and check additional components and optimize the vessel control system." (S5) "We cannot say, this is my part of the system, and I don’t care for the rest. This system works both in diesel and electric mode, it has software and propulsion components. All of these components need to be in harmony to not affect each other negatively."  (S7) "The realistic mode for me to provide energy is with the help of a diesel generator." |
| **Phase 5 New means and new goals** | | |
| **Characteristics** | **Key informant terms** | **Representative quotes from interviewees** |
| Expanding of resources | Resources expand through the collaborative innovation | (S5) "Engineering is monitoring what is being done on the market and that way we can contribute to new ideas." (S11) "In 2000, we put two electric motors for this purpose. Now we can choose either for the ship to be driven by two main engines or by two electrical motors, which take power from another big generator. This improved our ability to run the ship more flexibly." |
| Converging on goals | Goals of the innovation are converged with a clearer direction for further innovation | (S4) "Hybrid engine is still a process which is unfinished and it didn’t reach the final phase, where we could consider the project done. " (S9) "Seven years ago we had a reconstruction of the marina, and we planned to make a stronger electricity infrastructure in the marina." (S11) "The one big generator ensured 8 to 10 knots of speed. This is bringing us to save 15 to 20% of the fuel. We shift the range of the engine to the optimal power range. Now we are going to the new project, the sail assisting vessel. Energy can be saved only through gaining external energy or through spending less. " |
| **Knowledge absorption: Acquisition** | | |
| **Characteristics** | **Key informant terms** | **Representative quotes from interviewees** |
| Acquire value of external knowledge | Perception of uncertainty: anticipating the future | (S12) "I think that hybridization is useful to a certain extent in the upcoming years. However, in 2030, hybridization should be something in the past. We will have to have vessels which are not hybridized but have other renewable energy sources as fuel. " (S13) "I have doubts whether we should waste energy for synthetic fuel development, because it requires a lot of energy. Whether it is bio based or synthetic, like LNG or care, it requires a disproportionate amount of energy to produce that type of fuel. In a longer type we will skip this type of fuel. " |
|  | Need assessment: navigating challenges and opportunities | (S1)"Our main issue was the battery. The battery is a burden, which doesn’t change through time." (S1)"The weight doesn’t change whether the battery is full or empty. That is one issue." (S1) "Larger applications would definitely benefit from hybrid installations. Especially due to the new IMO regulations, which require the boat to run on zero-emissions once entering the port. So, it makes sense for larger vessels but not for smaller."  (S6) "When you start a business, you have a specific set of capabilities or interests. Then you try to specify a niche on the market. You see someone has a specific need or you try to push these needs, in the combination of these two the space for a company development is set." |
|  | Trend observation: shift towards green technologies | (S3)"Other segments of the market are not satisfied with 5 knots, they also want to be green, but they want to be faster. " (S3) "Hotels are now closed and people start buying trailer parks and boats, to not be in the crowd and cities. The electric has a place on the sea, but the customers have to be ready." (S11) "There is a huge pressure on shipping. The carbon footprint regulations are influencing all parts of the marine industry. " (S11) "Everybody thinks electric is a winner. Of course it is relatively, but it’s not the ultimate, unless it’s renewable energy." (S11) "There are many companies interested in hybrid propulsion." |
| **Knowledge absorption: Assimilation** | | |
| **Characteristics** | **Key informant terms** | **Representative quotes from interviewees** |
| Comprehend and internalize knowledge | Perceive competition as an opportunity | (S4) "The competition is growing because demand is growing. Consequently, the demand for hybrid systems is on the rise." (S11) "There is a competition starting at the moment. This is a new niche which just opened." (S11) "The competition is starting now. Already many companies are getting interested in hybrids. " |
|  | Evaluate and manage relevant risks | (S2) "Also, there is a logistic problem of acquiring, storing and charging the batteries. " (S9) "That is a potential danger for the marina, and we always view it from the risk point of view. We know very well that if a vessel is on fire, only the fire on the surrounding vessels is being put down, not the burning vessel." (S11) "Already there are a few cases in the fleets where batteries caught fire. The fire is the most dangerous. The batteries can burn even without oxygen, in a sealed compartment. The battery gives the opportunity to use excess energy gained from the propulsion." (S12) "Vessels which were built this or in the next two to three years, will be in motion at least until 2050. Decarbonization of the whole sector is needed to be almost completed by the middle of the century. In that sense, I don’t see hybridization as a long-term solution. In ten years, hybridization will be more a risky investment rather than a solution." (S13) "This is a direct carry over of the technology from the automotive industry and this vessel will never be certified by DMV due to safety regulations." |
|  | Advancements and challenges: analyze and assimilate new knowledge | (S2) "There are several prototypes. Most of the prototypes use fuel cells and not internal combustion engines. Fuel cells do have some disadvantages, regarding the slow start in working. " (S6) "This is of course not without a reason; you need to have the energy which you are carrying with yourself. Either be it in the form of batteries or if you are producing the energy yourself. On board, this is problematic. " (S6) "For boats, there are two questions. How to produce electric energy from hydrogen...The second problem is storage." |
| **Knowledge absorption: Transformation** | | |
| **Characteristics** | **Key informant terms** | **Representative quotes from interviewees** |
| Integrate knowledge to leverage innovation for opportunity | Retain existing knowledge and transform new knowledge | (S2) "The electric engine itself is constructionally less complex and less expensive than an engine with internal combustion, but for now the amount of energy required by the vessels can’t be met, depending on the case." (S2) "So far I believe that the electric and hybrid systems have been more developed for cars and they seem to be more feasible." (S12) "Of course, there are hybrid types where people can use hydrogen and an electric battery. A hybrid engine doesn’t need to necessarily use a diesel engine or LNG. " |
|  | Transform knowledge to grasp the identified opportunities | (S10) "About seventy percent of the yachts have solar panels on the top, but eighty to ninety have the shaft or hydro generator. That is because hydro generators are more efficient. While solar produce ten percent of the energy, hydro generators produce ninety percent of the energy. Solar panels are more to keep the batteries topped up, but not for the main consumption." (S13) "This vessel is sailing, but the first prototype for a small marine vessel is our boat." |
| **Knowledge absorption: Exploitation** | | |
| **Characteristics** | **Key informant terms** | **Representative quotes from interviewees** |
| Create value through established technology | Boost performance: harnessing established technology | (S5) "In technical application and engineering one can see and achieve potential possibilities for new improvements and ideas, which we have already done in the past." (S11) "The hybrid is not a magic stick solution. You don’t get renewable energy from the hybrid; you are still using diesel fuel to create electricity for propulsion. In the optimal way you are not wasting as much energy. " (S13) "One of the things that we learn is that we have to reorganize the architecture of the fuel cell, which is not easy. " (S13) "First of all, you cannot take a hydrogen fuel cell from the automotive industry and put it in a boat, that is due to safety regulations for boats. " |
|  | Integrate knowledge to create value: improving comfort and efficiency | (S3) "Before, *the brand* was designed for lakes and rivers, more inland waters with a single engine. It wasn’t designed for an open sea boat, but now we have to look at this solution." (S3) "All your life under diesel, vibration, noise, pollution and smell. But, on the hybrid boat you ride on the diesel, the batteries charge and when you stop, you start using the energy from the batteries. " (S10) "During those modes of operation, if you are under an engine, you can choose either an economic way of boating, where you motor just with the power of an electric motor or the diesel engine." |
